# Anatomic and Hemodynamic Plaque Characteristics for Subsequent Coronary Events

**DOI:** 10.3389/fcvm.2022.871450

**Published:** 2022-05-23

**Authors:** Seung Hun Lee, David Hong, Neng Dai, Doosup Shin, Ki Hong Choi, Sung Mok Kim, Hyun Kuk Kim, Ki-Hyun Jeon, Sang Jin Ha, Kwan Yong Lee, Taek Kyu Park, Jeong Hoon Yang, Young Bin Song, Joo-Yong Hahn, Seung-Hyuk Choi, Yeon Hyeon Choe, Hyeon-Cheol Gwon, Junbo Ge, Joo Myung Lee

**Affiliations:** ^1^Division of Cardiology, Department of Internal Medicine, Chonnam National University Hospital, Chonnam National University Medical School, Gwangju, South Korea; ^2^Division of Cardiology, Department of Internal Medicine, Heart Vascular Stroke Institute, Samsung Medical Center, Sungkyunkwan University School of Medicine, Seoul, South Korea; ^3^Department of Cardiology, Shanghai Institute of Cardiovascular Diseases, Zhongshan Hospital, Fudan University, Shanghai, China; ^4^Division of Cardiovascular Medicine, Department of Internal Medicine, University of Iowa Carver College of Medicine, Iowa City, IA, United States; ^5^Department of Radiology, Cardiovascular Imaging Center, Heart Vascular Stroke Institute, Samsung Medical Center, Sungkyunkwan University School of Medicine, Seoul, South Korea; ^6^Department of Internal Medicine and Cardiovascular Center, Chosun University Hospital, University of Chosun College of Medicine, Gwangju, South Korea; ^7^Division of Cardiovascular Medicine, Department of Internal Medicine, Seoul National University Bundang Hospital, Seongnam, South Korea; ^8^Division of Cardiology, Department of Internal Medicine, Gangneung Asan Hospital, University of Ulsan College of Medicine, Gangneung, South Korea; ^9^Cardiovascular Center and Cardiology Division, Seoul St. Mary's Hospital, The Catholic University of Korea, Seoul, South Korea

**Keywords:** coronary artery disease, coronary CT angiography, myocardial ischemia, vulnerable plaques, prognosis

## Abstract

**Objectives:**

While coronary computed tomography angiography (CCTA) enables the evaluation of anatomic and hemodynamic plaque characteristics of coronary artery disease (CAD), the clinical roles of these characteristics are not clear. We sought to evaluate the prognostic implications of CCTA-derived anatomic and hemodynamic plaque characteristics in the prediction of subsequent coronary events.

**Methods:**

The study cohort consisted of 158 patients who underwent CCTA with suspected CAD within 6–36 months before percutaneous coronary intervention (PCI) for acute myocardial infarction (MI) or unstable angina and age-/sex-matched 62 patients without PCI as the control group. Preexisting high-risk plaque characteristics (HRPCs: low attenuation plaque, positive remodeling, napkin-ring sign, spotty calcification, minimal luminal area <4 mm^2^, or plaque burden ≥70%) and hemodynamic parameters (per-vessel fractional flow reserve [FFRCT], per-lesion ΔFFR_CT_, and percent ischemic myocardial mass) were analyzed from prior CCTA. The primary outcome was a subsequent coronary event, which was defined as a composite of vessel-specific MI or revascularization for unstable angina. The prognostic impact of clinical risk factors, HRPCs, and hemodynamic parameters were compared between vessels with (160 vessels) and without subsequent coronary events (329 vessels).

**Results:**

Vessels with a subsequent coronary event had higher number of HRPCs (2.6 ± 1.4 vs. 2.3 ± 1.4, *P* = 0.012), lower FFR_CT_ (0.76 ± 0.13 vs. 0.82 ± 0.11, *P* < 0.001), higher ΔFFR_CT_ (0.14 ± 0.12 vs. 0.09 ± 0.08, *P* < 0.001), and higher percent ischemic myocardial mass (29.0 ± 18.5 vs. 26.0 ± 18.4, *P* = 0.022) than those without a subsequent coronary event. Compared with clinical risk factors, HRPCs and hemodynamic parameters showed higher discriminant abilities for subsequent coronary events with ΔFFR_CT_ being the most powerful predictor. HRPCs showed additive discriminant ability to clinical risk factors (c-index 0.620 vs. 0.558, *P* = 0.027), and hemodynamic parameters further increased discriminant ability (c-index 0.698 vs. 0.620, *P* = 0.001) and reclassification abilities (NRI 0.460, IDI 0.061, *P* < 0.001 for all) for subsequent coronary events. Among vessels with negative FFR_CT_ (>0.80), adding HRPCs into clinical risk factors significantly increased discriminant and reclassification abilities for subsequent coronary events (c-index 0.687 vs. 0.576, *P* = 0.005; NRI 0.412, *P* = 0.002; IDI 0.064, *P* = 0.001) but not for vessels with positive FFR_CT_ (≤0.80).

**Conclusion:**

In predicting subsequent coronary events, both HRPCs and hemodynamic parameters by CCTA allow better prediction of subsequent coronary events than clinical risk factors. HRPCs provide more incremental predictability than clinical risk factors alone among vessels with negative FFR_CT_ but not among vessels with positive FFR_CT_.

**Clinical Trial Registration:**

PreDiction and Validation of Clinical CoursE of Coronary Artery DiSease With CT-Derived Non-INvasive HemodYnamic Phenotyping and Plaque Characterization (DESTINY Study), NCT04794868.

## Introduction

Identification of patients with coronary atherosclerotic disease (CAD) at high risk of acute coronary syndrome (ACS) and who may benefit from intensified preventive measures has been of major interest (1). Since postmortem studies provided insights into plaque vulnerability and rupture as the major causes of ACS and sudden cardiac death, various imaging modalities have been used to identify characteristics of vulnerable plaques, which are prone to rupture (high-risk plaque characteristics [HRPCs]) (2–6).

Nevertheless, given the limited predictive value of HRPCs alone in the prediction of subsequent coronary events, (7) contemporary practice has been guided by the hemodynamic significance of CAD determined by invasive physiologic indexes, such as fractional flow reserve (FFR) (8), but not by HRPCs. Recent studies have suggested the importance of both hemodynamic significance and plaque vulnerability in CAD and their complementary roles in the progression of the disease and the development of ACS (9). Comprehensive assessment of CAD has become possible in clinical practice with recent advances in coronary computed tomography angiography (CCTA) and computational fluid dynamics (CFD), which allow simultaneous noninvasive assessment of anatomic plaque characteristics (4–6) and hemodynamic significance of CAD (10).

However, studies on the clinical role of the comprehensive evaluation of these features are limited, and it is still unclear whether integrating various aspects of the pathophysiology of CAD would increase the predictability of subsequent coronary events (9). Furthermore, it would be important to better understand the prognostic implications and the potential role of utilizing HRPCs in contemporary CAD management. In this regard, this study sought to evaluate (1) prognostic implications of combined analysis of CCTA-derived HRPCs and hemodynamic parameters in the prediction of subsequent coronary events and (2) differential prognostic implications of CCTA-derived HRPCs according to the hemodynamic significance of CAD.

## Materials and Methods

### Study Design and Population

**Figure 1:**
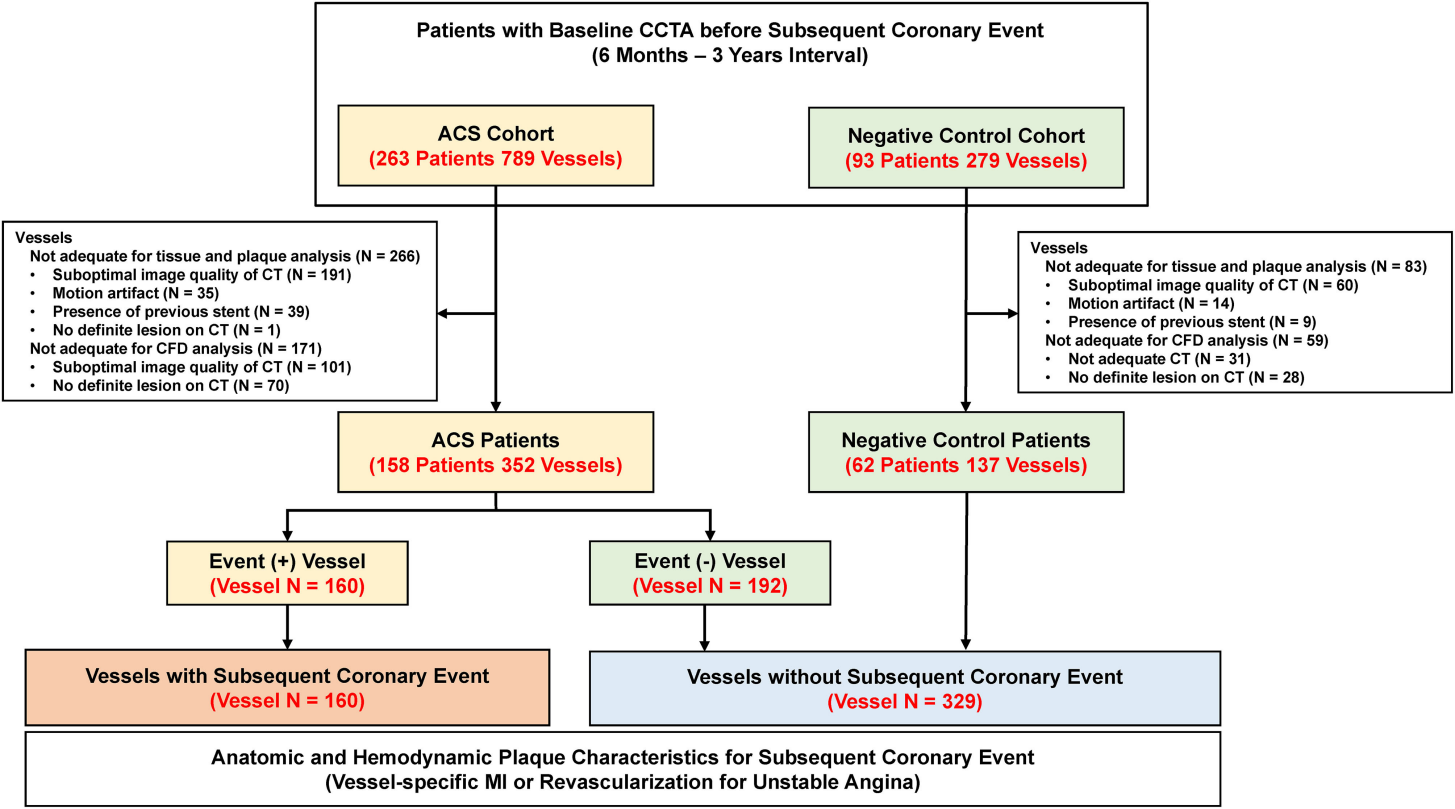
Study flow. This study enrolled two separate patient populations (ACS and negative control cohort). The ACS cohort included consecutive patients who underwent CCTA within 6–36 months before PCI for acute myocardial infarction or unstable angina. The negative control cohort included age-/sex-matched patients who underwent CCTA within 6–36 months before invasive coronary angiography for suspected stable angina but did not undergo PCI because there was no significant lesion at the time of angiography. A total of 489 vessels (160 vessels with subsequent coronary event and 329 vessels without subsequent coronary event) were finally included in the study. ACS, acute coronary syndrome; CCTA, coronary computed tomography angiography; CFD, computational fluid dynamics; CT, computed tomography; PCI, percutaneous coronary intervention.

To evaluate the prognostic impact of anatomic and hemodynamic plaque characteristics on subsequent coronary events, this study enrolled two separate patient populations (the ACS cohort and the negative control cohort) (Figure 1). The ACS cohort included consecutive patients who underwent CCTA within 6–36 months before percutaneous coronary intervention (PCI) for acute myocardial infarction (MI) or unstable angina admitted to Samsung Medical Center between 2003 and 2019. The negative control cohort included age-/sex-matched patients who underwent CCTA within 6–36 months before invasive coronary angiography for suspected stable angina but did not undergo PCI because there was no significant lesion at the time of angiography. In both cohorts, CCTA was performed under the judgment of the respective physicians as a routine clinical evaluation for suspected CAD. In addition, the operators were blinded to the detailed core laboratory analyses of anatomic and hemodynamic plaque characteristics at the time of invasive angiography.

In the ACS cohort, patients without clear culprit lesions in invasive angiography, intravascular ultrasound, or optical coherence tomography were excluded. Additional exclusion criteria were patients with ACS caused by in-stent restenosis, vessels with stents before CCTA, previous history of coronary artery bypass grafting, and type 2 myocardial infarction due to other general medical conditions. In both ACS and negative control cohorts, patients with unavailable CCTA images or suboptimal image quality for the analysis of plaque characteristics or CFD were excluded by the CCTA core laboratory (Elucid Bioimaging, Inc., Boston, MA, USA) or the CFD core laboratory (Shanghai Institute of Cardiovascular Diseases, Shanghai, China), respectively. The study protocol was approved by the institutional review board of Samsung Medical Center. This study was conducted in accordance with the Declaration of Helsinki and registered on clinicaltrials.gov (PreDiction and Validation of Clinical CoursE of Coronary Artery DiSease With CT-Derived Non-INvasive HemodYnamic Phenotyping and Plaque Characterization [DESTINY Study], NCT02374775).

### Analysis of Anatomic Plaque Characteristics in CCTA

Coronary computed tomography angiography images were obtained in accordance with the Society of Cardiovascular Computed Tomography Guidelines on Performance of CCTA, with 64-channel scanner (GE Healthcare, Milwaukee, WI, USA) or 128-channel dual-source scanner platforms (Siemens Medical System, Forchheim, Germany) with electrocardiographic gating (11). A standardized protocol for heart rate control with beta-blockers and sublingual nitroglycerin was administered. All CCTA images were analyzed regarding anatomic plaque characteristics in a blinded fashion using histologically validated plaque quantification software (vascuCAP, clinical edition) at a core laboratory (Elucid Bioimaging, Inc., Boston, MA, USA) (12).

For anatomic severity, diameter stenosis, area stenosis, minimum lumen area (MLA), and lesion length were measured. Whole vessel and plaque tissue characterization were performed by defining the vessel wall into different components: calcified tissue, intra-plaque hemorrhage, lipid-rich necrotic core, matrix, or perivascular adipose tissue (13). Plaque burden was defined as the ratio of wall area divided by the overall vessel area (12, 13). Any lesions with diameter stenosis >30% were selected for plaque tissue characterization. In cases of multiple lesions in the same target vessel, the lesion with the greatest diameter stenosis was selected as the representative lesion. The presence of the following HRPCs was analyzed according to the definitions from previous studies: (1) low attenuation plaque (average density ≤ 30 Hounsfield units [HU].); (2) positive remodeling (lesion diameter/reference diameter ≥1.1); (3) napkin-ring sign (ring-like attenuation pattern with peripheral high attenuation tissue surrounding a central lower attenuation portion); and (4) spotty calcification (average density >130 HU and diameter <3 mm) (4, 5). In this analysis, HRPCs were defined by combining both qualitative and quantitative parameters based on previous studies with the presence of any of the following features: low attenuation plaque, positive remodeling, napkin-ring sign, spotty calcification, MLA <4 mm^2^, or plaque burden ≥70% (2–5).

### Analysis of Hemodynamic Plaque Characteristics in CCTA

Hemodynamic parameters derived from CCTA were analyzed in a blinded fashion at a core laboratory (Zhongshan Hospital, Shanghai, China) using a commercialized offline software system (RuiXin-FFR, version 1.0, Raysight Medical, Shenzhen, China). First, three-dimensional anatomical computational models of the coronary tree were reconstructed from CCTA images. Second, patient-specific boundary conditions were obtained from the CCTA images. Third, hemodynamics parameters were solved by CFD-based FFR_CT_ calculation. Detailed methods of three-dimensional model reconstruction and CFD-based FFR_CT_ calculation are described in the Supplementary Appendix. Briefly, coronary models were constructed using segmentation algorithms that extracted the luminal surface of the epicardial coronary arteries and branches. Coronary flow and pressure were computed by solving the Navier–Stokes equations, assuming that blood is approximated as a Newtonian fluid. Boundary conditions for hyperemia were derived from myocardial mass, vessel sizes at each outlet, and the response of the microcirculation to adenosine. As with plaque tissue characterization, only lesions with a diameter stenosis of >30% were selected for the computation of hemodynamic parameters. For this study, three hemodynamic parameters were used, namely, per-vessel FFR_CT_, per-lesion delta FFR_CT_ (ΔFFR_CT_), and per-vessel percent ischemic myocardial mass. First, FFR_CT_ was defined as the ratio of mean downstream coronary pressure (*P*_d_) and the aortic pressure (*P*_a_) derived from the CFD analysis under a simulated hyperemic condition. Second, ΔFFR_CT_ was defined by computing the difference in FFR_CT_ values at the proximal and distal sites of each lesion. Third, the percent ischemic myocardial mass of each vessel segment was defined as the ratio between the myocardial mass subtended beyond the point at which the vessel's FFR_CT_ is ≤0.80 and the entire vessel segment (14). Myocardial mass was computed using a stem-and-crown model (15, 16), which is based on allometric scaling between the length of the coronary arterial tree and myocardial mass (15, 16).

### Data Collection and Clinical Outcomes

Clinical data were collected by reviewing electronic medical records. All angiograms were analyzed, and culprit lesions were determined in a blinded fashion at core laboratories (Samsung Medical Center, Seoul, Korea). The primary outcome was a subsequent coronary event, which was defined as a composite of vessel-specific MI or revascularization for unstable angina. The definition of clinical outcomes was in accordance with the Academic Research Consortium. Acute MI was defined according to the universal definition of MI (17).

### Statistical Analysis

Data were analyzed on a per-patient basis for clinical characteristics and on a per-vessel basis for anatomic and hemodynamic plaque characteristics and vessel-specific clinical outcomes. For per-patient analyses, the Student's *t*-test and the chi-square test were used to compare continuous and categorical variables, respectively. For per-vessel analyses, a generalized estimating equation was used to adjust for intra-subject variability among vessels from the same patient. An analysis of variance test was used to compare differences in the number of clinical risk factors and hemodynamic parameters according to the classification by the number of HRPCs.

The discriminant function of clinical characteristics and anatomic and hemodynamic plaque characteristics for the primary outcome were evaluated using the c-index and 95% confidence interval (CI) in receiver operating curve analysis. Optimal cutoff values for HRPCs and hemodynamic parameters were determined based on receiver operating curve analysis and results of previous studies (5, 8, 9). Diagnostic performance was presented as sensitivity, specificity, positive predictive value, negative predictive value, and diagnostic accuracy. Incremental predictability of HRPCs and hemodynamic parameters for the primary outcome was compared using a global chi-square estimated by the likelihood ratio test. The cumulative incidence of the primary outcome was presented as Kaplan–Meier estimates and compared using a log-rank test. To adjust for the interrogated vessels within the same patient, multivariable marginal Cox proportional hazards regression was used to calculate the adjusted hazard ratio (HR) and 95% CI. Adjusted covariables were age, sex, hypertension, diabetes mellitus, dyslipidemia, chronic kidney disease, and current smoker. The assumption of proportionality was assessed graphically using a log minus log plot, and all Cox proportional hazard models satisfied the proportional hazards assumption.

Three prediction models were constructed to assess the incremental prognostic value of HRPCs and hemodynamic parameters: (1) model 1: clinical risk factors; (2) model 2: model 1 + individual components of HRPCs; and (3) model 3: model 2 + hemodynamic parameters. Clinical risk factors included age, sex, hypertension, diabetes mellitus, dyslipidemia, chronic kidney disease, and current smoker. Hemodynamic parameters included FFR_CT_, ΔFFR_CT_, and percent ischemic myocardial mass. Discriminant ability was compared using the c-index, and reclassification performance was compared using the relative integrated discrimination improvement (IDI) and category-free net reclassification index (NRI). Subgroup analysis was performed to assess the differential prognostic implications of HRPCs according to hemodynamic significance. Vessels were divided into subgroups according to optimal cutoff values of FFR_CT_ and ΔFFR_CT_, and the incremental prognostic value for the primary outcome of HRPCs was evaluated in each subgroup.

All analyses were two-sided, and *P-*values <0.05 were considered statistically significant. Statistical analyses were performed using R version 4.0.3 (R Foundation for Statistical Computing, Vienna, Austria).

## Results

### Characteristics of Patients

A total of 220 patients with 489 vessels were selected for the current analyses (Figure 1). Among them, 158 patients (71.8%) were from the ACS cohort and 62 (28.2%) were from the negative control cohort. In the ACS cohort, 17.1 and 82.9% of patients presented with acute MI and unstable angina, respectively. Among the ACS cohort, 160 vessels had subsequent coronary events and 192 vessels were non-culprit vessels. With 137 vessels from the negative control cohort, a total of 329 vessels were not related to subsequent coronary events. The mean interval between CCTA and invasive coronary angiography was 554.3 ± 268.2 days. In the comparison of the clinical characteristics of patients, there was no significant difference in demographics, cardiovascular comorbidities, or profiles of medical treatment after CCTA (Table 1).

**Table 1:** Baseline clinical characteristics.

**Variables**	**Total patient (*N =* 220)**	**ACS patient (*N =* 158)**	**Negative control patient (*N =* 62)**	***P*-value**
**Demographics**
Age, years	65.5 ± 10.2	65.2 ± 10.6	66.2 ± 9.3	0.488
Men	179 (81.4)	131 (82.9)	48 (77.4)	0.454
CCTA—ICA Interval	554.3 ± 268.2	535.7 ± 260.7	601.9 ± 282.9	0.100
**Clinical presentation**				<0.001
ST-segment elevation myocardial infarction	10 (4.5)	10 (6.3)	0 (0.0)	
Non-ST-segment elevation myocardial infarction	17 (7.7)	17 (10.8)	0 (0.0)	
Unstable angina	131 (59.5)	131 (82.9)	0 (0.0)	
Stable angina	62 (28.2)	0 (0.0)	62 (100.0)	
**Cardiovascular risk factors**
Hypertension	160 (72.7)	113 (71.5)	47 (75.8)	0.635
Diabetes mellitus	131 (59.5)	94 (59.5)	37 (59.7)	>0.999
Dyslipidemia	93 (42.3)	63 (39.9)	30 (48.4)	0.318
Chronic kidney disease	15 (6.8)	11 (7.0)	4 (6.5)	>0.999
Current smoker	42 (19.1)	30 (19.0)	12 (19.4)	>0.999
History of percutaneous coronary intervention	25 (11.4)	24 (15.2)	1 (1.6)	0.009
History of myocardial infarction	12 (5.5)	10 (6.3)	2 (3.2)	0.561
History of cerebrovascular accident	34 (15.5)	24 (15.2)	10 (16.1)	>0.999
History of peripheral vascular disease	14 (6.4)	9 (5.7)	5 (8.1)	0.734
**Medical treatment after CCTA before clinical event**
Antiplatelet agent	167 (75.9)	123 (77.8)	44 (71.0)	0.369
ACEI or ARB	100 (45.5)	70 (44.3)	30 (48.4)	0.692
Beta blocker	81 (36.8)	59 (37.3)	22 (35.5)	0.919
Calcium channel blocker	84 (38.2)	59 (37.3)	25 (40.3)	0.799
Statin	146 (66.4)	104 (65.8)	42 (67.7)	0.910
Ezetimibe	8 (3.6)	7 (4.4)	1 (1.6)	0.546
**Echocardiographic findings**
Left ventricular ejection fraction, %	62.2 ± 9.5	61.9 ± 9.9	63.2 ± 8.5	0.428

*Data are presented as mean ± standard deviation or number (%)*.

*ACEI, angiotensin-converting enzyme inhibitor; ACS, acute coronary syndrome; ARB, angiotensin receptor blocker; CCTA, coronary computed tomography angiography; ICA, invasive coronary angiography*.

### Anatomic and Hemodynamic Plaque Characteristics

Table 2 shows the comparison of anatomic and hemodynamic plaque characteristics between 160 vessels with subsequent coronary events and 329 vessels without subsequent coronary events. In addition, Supplementary Table 1 presents the anatomical and hemodynamic plaque characteristics of 137 vessels from the negative control cohort. Vessels with subsequent coronary events showed significantly lower MLA and higher plaque burden than vessels without events. Regarding anatomic plaque characteristics, vessels with subsequent coronary events showed a significantly higher proportion of low attenuation plaque, MLA <4 mm^2^, and plaque burden at lumen area ≥70% than vessels without events. As a result, vessels with subsequent coronary events had a higher number of HRPCs (2.6 ± 1.4 vs. 2.3 ± 1.4, *P* = 0.009) than vessels without events. There was no significant difference in other individual components of the whole vessel and plaque tissue characterization between the two groups.

**Table 2:** Anatomic and hemodynamic plaque characteristics.

**Variables**	**Total Vessels (*N =* 489)**	**Vessels with subsequent coronary event (*N =* 160)**	**Vessels without subsequent coronary event (*N =* 329)**	***P*-value**
**Interrogated Vessels**				0.002
Left anterior descending artery	189 (38.7)	79 (49.4)	110 (33.4)	
Left circumflex artery	143 (29.2)	35 (21.9)	108 (32.8)	
Right coronary artery	157 (32.1)	46 (28.8)	111 (33.7)	
**Anatomical severity**
Diameter stenosis, %	54.4 ± 18.2	58.4 ± 18.2	52.6 ± 17.9	0.001
Area stenosis, %	63.1 ± 19.3	66.6 ± 19.0	61.4 ± 19.3	0.006
Minimum lumen area, mm^2^	1.8 ± 1.4	1.5 ± 1.2	1.9 ± 1.4	0.002
Lesion length, mm	22.5 ± 17.2	24.4 ± 16.9	21.6 ± 17.3	0.175
**Whole vessel tissue characterization**
Plaque burden, %	80.2 ± 12.6	82.9 ± 11.5	78.9 ± 13.0	0.001
Calcified volume, %	4.3 ± 5.5	4.4 ± 5.3	4.3 ± 5.6	0.919
Maximum calcified area, %	24.8 ± 22.4	24.4 ± 20.9	25.0 ± 23.1	0.781
Intra-plaque hemorrhage volume, mm^3^	4.2 ± 7.8	4.8 ± 10.0	4.0 ± 6.4	0.374
Maximum intra-plaque hemorrhage area, mm^2^	0.73 ± 0.96	0.77 ± 0.91	0.71 ± 0.99	0.565
Lipid-rich necrotic core volume, mm^3^	1.4 ± 3.9	1.9 ± 5.0	1.2 ± 3.2	0.150
Maximum lipid-rich necrotic core area, mm^2^	0.35 ± 0.68	0.41 ± 0.77	0.32 ± 0.63	0.202
Perivascular adipose tissue volume, %	30.2 ± 12.6	31.8 ± 12.4	29.4 ± 12.6	0.060
Vessel length, mm	75.2 ± 35.4	75.2 ± 33.8	75.2 ± 36.2	0.984
**Target plaque tissue characterization**
Plaque burden, %	82.2 ± 11.9	85.5 ± 9.9	80.5 ± 12.5	<0.001
Calcified volume, %	11.6 ± 8.1	10.6 ± 8.2	12.1 ± 8.1	0.119
Maximum calcified area, %	32.8 ± 20.9	31.1 ± 20.1	33.6 ± 21.3	0.278
Intra-plaque hemorrhage volume, mm^3^	2.3 ± 4.6	3.1 ± 6.2	2.0 ± 3.5	0.087
Maximum intra-plaque hemorrhage area, mm^2^	0.60 ± 0.87	0.76 ± 1.03	0.52 ± 0.77	0.037
Lipid-rich necrotic core volume, mm^3^	1.2 ± 3.6	1.7 ± 5.1	1.0 ± 2.5	0.179
Maximum lipid-rich necrotic core area, mm^2^	0.31 ± 0.63	0.36 ± 0.71	0.29 ± 0.58	0.363
**High-risk plaque characteristics**
Low attenuation plaque	102 (20.9)	46 (28.8)	56 (17.0)	0.004
Positive remodeling	211 (43.1)	75 (46.9)	136 (41.3)	0.288
Napkin-ring sign	80 (16.4)	27 (16.9)	53 (16.1)	0.933
Spotty calcification	334 (68.3)	113 (70.6)	221 (67.2)	0.505
Minimum lumen area <4mm^2^	401 (82.0)	140 (87.5)	261 (79.3)	0.037
Plaque burden at lumen area ≥70%	29 (5.9)	16 (10.0)	13 (4.0)	0.014
Number of HRPCs	2.4 ± 1.4	2.6 ± 1.4	2.3 ± 1.4	0.009
Number of HRPCs ≥3	236 (48.3)	92 (57.5)	144 (43.8)	0.006
**Hemodynamic plaque characteristics**
FFR_CT_	0.80 ± 0.12	0.76 ± 0.13	0.82 ± 0.11	<0.001
ΔFFR_CT_	0.11 ± 0.10	0.14 ± 0.12	0.09 ± 0.08	<0.001
Ischemia myocardial mass, %	26.9 ± 18.5	29.0 ± 18.5	26.0 ± 18.4	0.022

*Data are presented as mean ± standard deviation, median (Q1–Q3), or number (%)*.

*FFR_CT_, fractional flow reserve by coronary computed tomography angiography; HRPCs, high-risk plaque characteristics*.

In terms of hemodynamic plaque characteristics, vessels with subsequent coronary events had lower FFR_CT_ (0.76 ± 0.13 vs. 0.82 ± 0.11, *P* < 0.001), higher ΔFFR_CT_ (0.14 ± 0.12 vs. 0.09 ± 0.08, *P* < 0.001), and higher percent ischemic myocardial mass (29.0 ± 18.5 vs. 26.0 ± 18.4, *P* = 0.022) than vessels without events. There was a significant association between the number of HRPCs and the number of clinical risk factors, FFR_CT_, ΔFFR_CT_, and percent ischemic myocardial mass. With an increased number of HRPCs, there was a significant increase in the number of clinical risk factors, ΔFFR_CT_, and percent ischemic myocardial mass and a significant decrease in FFR_CT_ (overall *P* < 0.001 for all comparisons) (Supplementary Figure 1).

### Prognostic Implications of Individual Anatomic and Hemodynamic Plaque Characteristics

**Figure 2:**
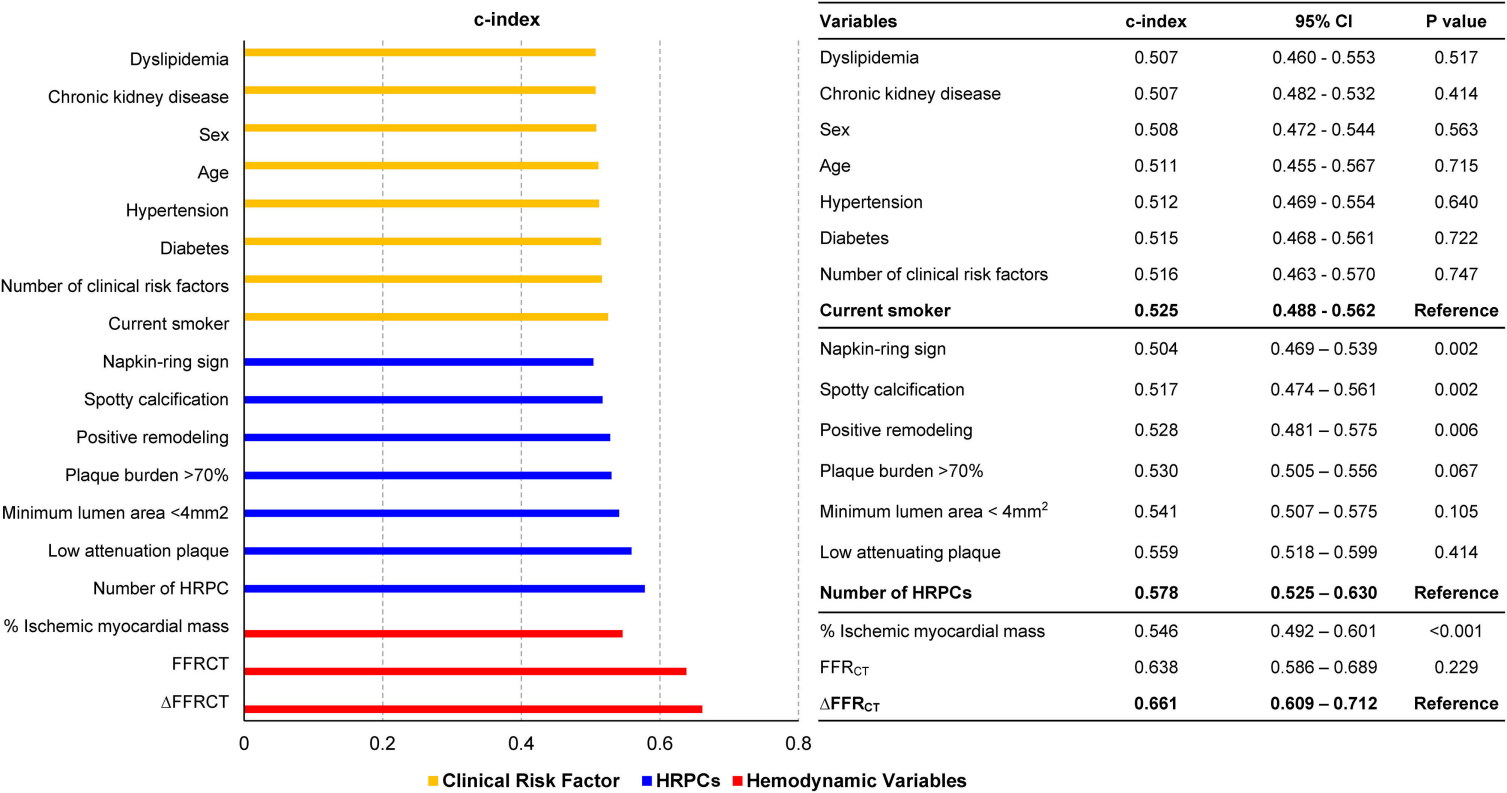
Comparison of discrimination abilities of clinical risk factors, HRPCs, and hemodynamic parameters for subsequent coronary events. Discrimination abilities of clinical risk factors (green bar), HRPCs (blue bar), and hemodynamic parameters (red bar) for subsequent coronary events are presented as c-index and 95% CI. For various variables in each group, the c-index comparison was performed with the variable with the highest c-index in each group. CI, confidence interval; FFR_CT_, fractional flow reserve by coronary computed tomography angiography; HRPCs, high-risk plaque characteristics.

Compared with clinical risk factors, individual components of HRPCs and hemodynamic parameters showed higher discriminant abilities for subsequent coronary events. Among anatomic and hemodynamic parameters, ΔFFR_CT_ showed the highest c-index to predict subsequent coronary events (c-index 0.661, 95% CI: 0.609–0.712) (Figure 2).

**Figure 3:**
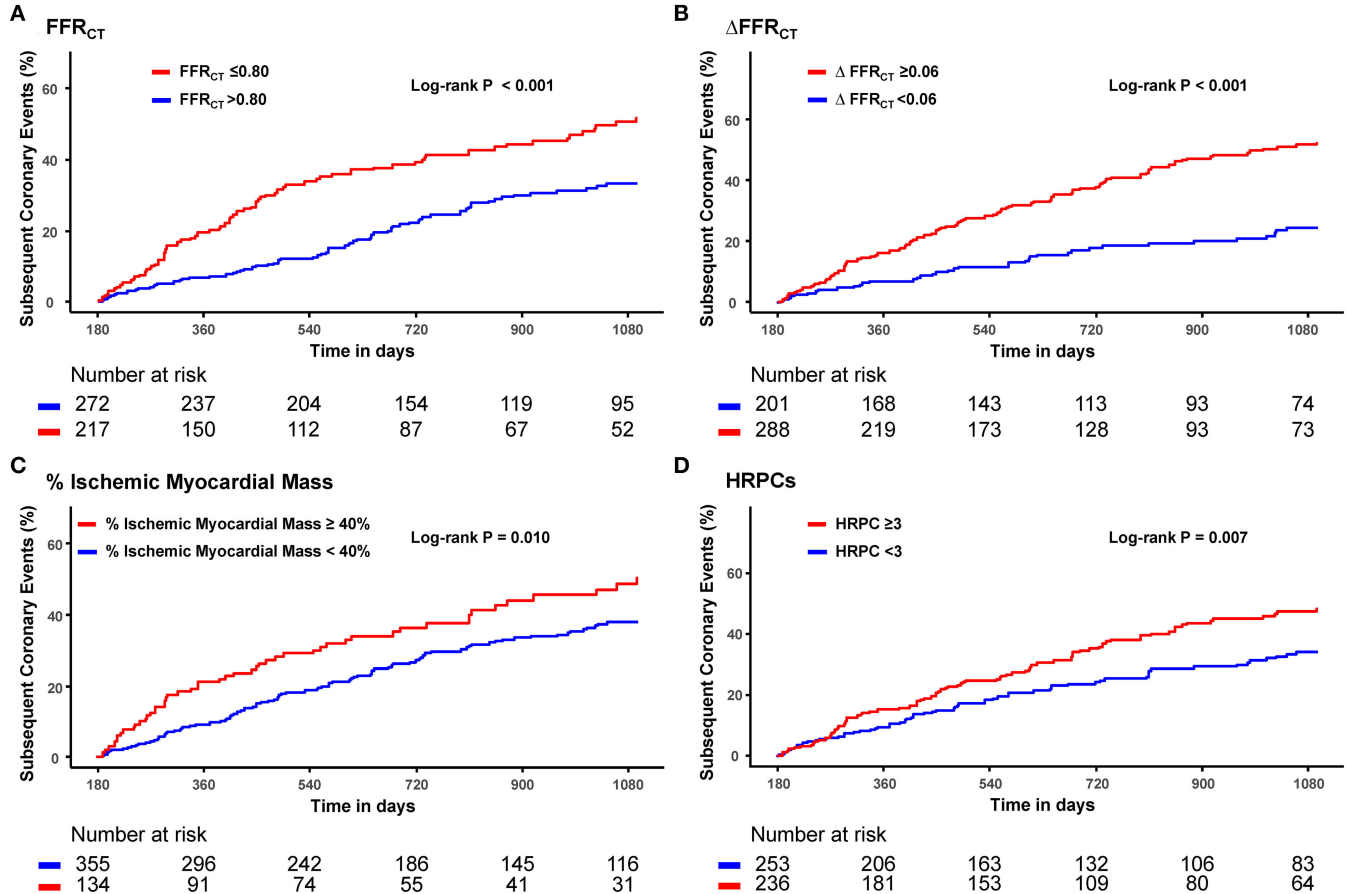
Cumulative incidence of subsequent coronary events according to FFR_CT_, ΔFFR_CT_, % ischemic myocardial mass, and HRPCs. Cumulative incidence of subsequent coronary events according to optimal cutoff values of **(A)** FFR_CT_, **(B)** ΔFFR_CT_, **(C)** % ischemic myocardial mass, and **(D)** HRPCs. FFR_CT_, fractional flow reserve by coronary computed tomography angiography; HRPCs, high-risk plaque characteristics.

Optimal cutoff values of hemodynamic parameters and the number of HRPCs for predicting subsequent coronary events were FFR_CT_ ≤ 0.80, ΔFFR_CT_ ≥ 0.06, percent ischemic myocardial mass ≥ 40%, and the number of HRPCs ≥ 3 (Supplementary Table 2). When the risk of subsequent coronary events was compared according to optimal cutoff values of hemodynamic parameters and the number of HRPCs, vessels with FFR_CT_ ≤ 0.80, ΔFFR_CT_ ≥ 0.06, percent ischemic myocardial mass ≥40%, and the number of HRPCs ≥3 were independently associated with an increased risk of subsequent coronary events than vessels with FFR_CT_ > 0.80, ΔFFR_CT_ < 0.06, percent ischemic myocardial mass <40%, and the number of HRPCs <3, respectively (Figure 3 and Table 3).

**Table 3:** Cumulative incidence of subsequent coronary events according to hemodynamic parameters and HRPCs.

**Variables**	**Cumulative Incidence** ^*^		**Unadjusted HR (95% CI)**	***P*-value**	**Adjusted HR^†^ (95% CI)**	***P*-value**
**FFR**_**CT**_	**≤0.80**	51.7% (88)	1.96 (1.44–2.68)	<0.001	2.05 (1.49–2.82)	<0.001
	**>0.80**	33.2% (72)				
**ΔFFR**_**CT**_	**≥0.06**	52.1% (122)	2.61 (1.81–3.76)	<0.001	2.75 (1.94–3.89)	<0.001
	**<0.06**	24.5% (38)				
**% Ischemic myocardial mass**	**≥40**	50.3% (52)	1.54 (1.10–2.14)	0.011	1.59 (1.14–2.21)	0.006
	**<40**	37.8% (108)				
**HRPCs**	**≥3**	48.3% (92)	1.54 (1.13–2.11)	0.007	1.59 (1.16–2.18)	0.004
	**<3**	34.2% (68)				

*Values are % (n) unless otherwise indicated*.

* *Cumulative incidence of clinical outcomes presented as Kaplan–Meier estimates*.

† *Adjusted variables in the multivariable marginal Cox regression model were age, sex, hypertension, diabetes, dyslipidemia, chronic kidney disease, and current smoker*.

*CI, confidence interval; FFR_CT_, fractional flow reserve by coronary computed tomography angiography; HR, hazard ratio; HRPCs, high-risk plaque characteristics*.

**Figure 4:**
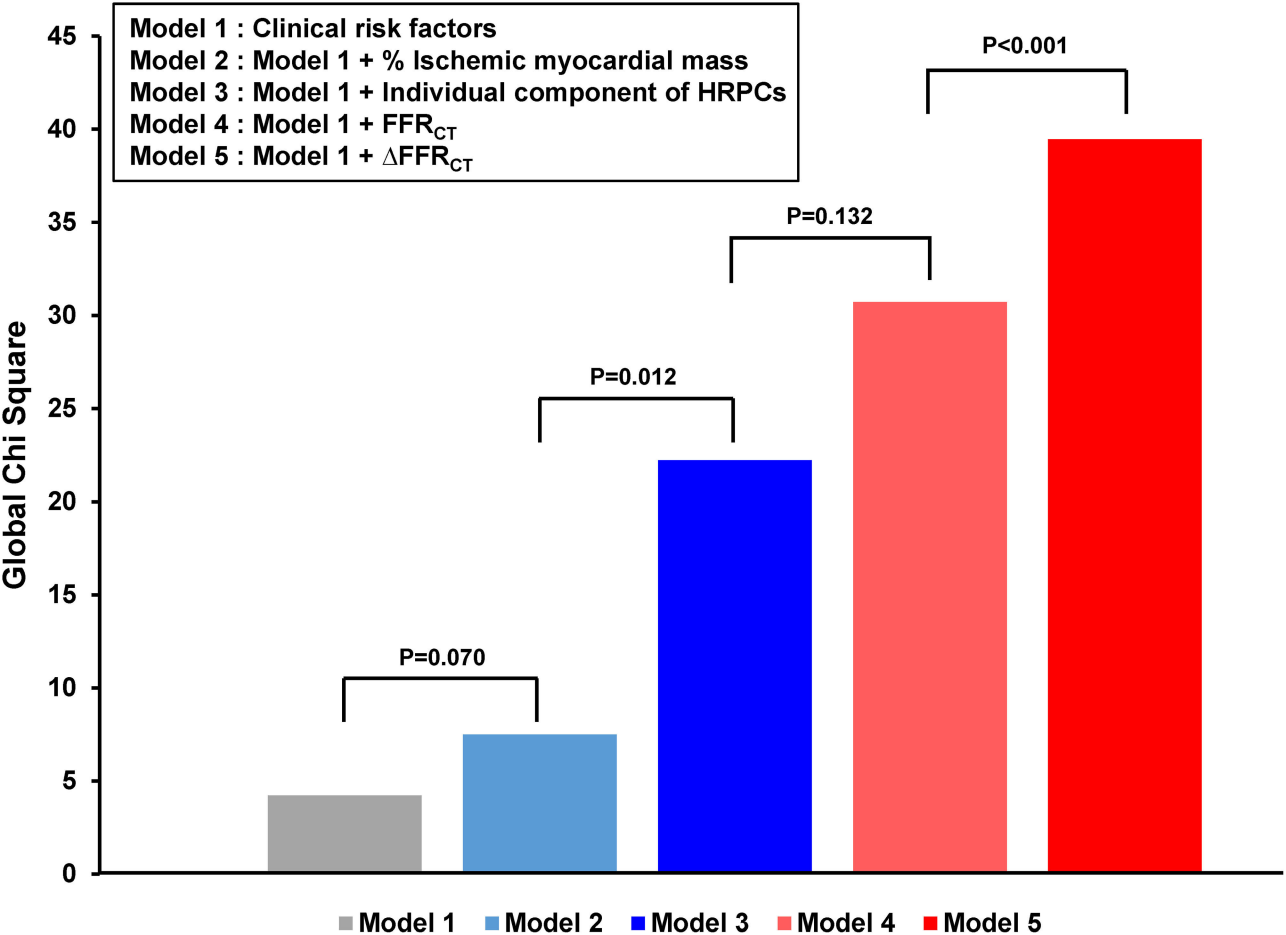
Incremental prognostic impact of HRPCs and hemodynamic parameters for subsequent coronary events. The addition of percent ischemic myocardial mass, HRPCs, ΔFFR_CT_, and ΔFFR_CT_ into clinical risk factors showed increased predictability for subsequent coronary events. Clinical risk factors included age, sex, hypertension, diabetes mellitus, dyslipidemia, chronic kidney disease, and current smoker. HRPCs included low attenuation plaque, positive remodeling, napkin-ring sign, spotty calcification, minimum luminal area <4 mm^2^, or plaque burden ≥70%. FFR_CT_, fractional flow reserve by coronary computed tomography angiography; HRPCs, high-risk plaque characteristics.

However, the positive predictive value and diagnostic accuracy of each hemodynamic parameter and the number of HRPCs were modest to predict subsequent coronary events as individual parameters (Supplementary Table 2). Nevertheless, the addition of percent ischemic myocardial mass, HRPCs, FFR_CT_, and ΔFFR_CT_ into clinical risk factors showed a stepwise increase in predictability for subsequent coronary events (Figure 4). Among anatomic and hemodynamic parameters, ΔFFR_CT_ showed the highest incremental predictability of the other variables (*P* < 0.001 for comparisons with the others).

### Prediction Models for Subsequent Coronary Events

Table 4 and Supplementary Figure 2 show the comparison of discriminant and reclassification abilities of three models for the prediction of subsequent coronary events. Compared with model 1 with clinical risk factors, additional integration of HRPCs into model 1 (model 2) showed higher discriminant ability (c-index 0.620 vs. 0.558, *P* = 0.027) and higher reclassification ability (NRI 0.269, *P* = 0.004; IDI 0.037, *P* < 0.001). Model 3, which included additional integration of hemodynamic parameters in model 2, further improved model 2 in terms of discriminant ability (c-index 0.698 vs. 0.620, *P* = 0.001) and reclassification ability (NRI 0.460, *P* < 0.001; IDI 0.061, *P* < 0.001) (Table 4 and Supplementary Figure 2). Furthermore, a simplified model (c-index 0.680, 95% CI: 0.630–0.731) was constructed by selecting only the variables with the best discriminant abilities among HRPCs (low attenuating plaque) and hemodynamic variables (ΔFFR_CT_), respectively, and adding them to clinical risk factors showed similar discriminant ability to model 3 (c-index 0.680 vs. 0.698, *P* = 0.234).

**Table 4:** Comparison of prediction models for subsequent coronary events.

Models^*^	**c-index**	**Difference with previous model**				
		**c-index comparison *P*-value**	**NRI**	***P*-value**	**IDI**	***P*-value**
Model 1	0.558 (0.504–0.611)					
Model 2	0.620 (0.566–0.674)	0.027	0.269 (0.084–0.455)	0.004	0.037 (0.019–0.055)	<0.001
Model 3	0.698 (0.648–0.747)	0.001	0.460 (0.277–0.644)	<0.001	0.061 (0.036–0.085)	<0.001

* *Models are constructed as follows: model 1: clinical risk factors; model 2: model 1 + individual component of HRPCs; and model 3: model 2 + hemodynamic parameters*.

*Components of clinical risk factors, hemodynamic parameters, and HRPCs are as follows: Clinical risk factors: age, sex, hypertension, diabetes, dyslipidemia, chronic kidney disease, and current smoker; Hemodynamic parameters: % ischemic myocardial mass, FFR_CT_, and ΔFFR_CT_; HRPCs: low attenuation plaque, positive remodeling, napkin-ring sign, spotty calcification, minimum lumen area <4 mm^2^, and plaque burden at lumen area ≥70%*.

*FFR_CT_, fractional flow reserve by coronary computed tomography angiography; HRPCs, high-risk plaque characteristics; IDI, relative integrated discrimination improvement; NRI, category-free net reclassification index*.

### Differential Prognostic Implication of HRPCs According to Hemodynamic Significance

To evaluate the differential prognostic implications of HRPCs according to hemodynamic significance, target vessels were stratified according to their hemodynamic significance determined by the optimal cutoff value of FFR_CT_ ≤ 0.80 vs. >0.80 or ΔFFR_CT_ ≥ 0.06 vs. <0.06. In vessels with FFR_CT_ ≤ 0.80, there was no significant difference in the distribution of HRPCs among vessels with or without subsequent coronary events. Conversely, in vessels with FFR_CT_ > 0.80, the vessels with subsequent coronary events showed a significantly higher proportion of low attenuating plaque and plaque burden ≥70% and showed a higher number of HRPCs (Supplementary Table 3). Stratified analysis by ΔFFR_CT_ showed similar results, and the number of HRPCs was significantly higher in vessels with subsequent coronary events only among vessels with ΔFFR_CT_ <0.06. Conversely, there was no significant difference in the number of HRPCs among vessels with ΔFFR_CT_ ≥ 0.06 (Supplementary Table 4).

**Figure 5:**
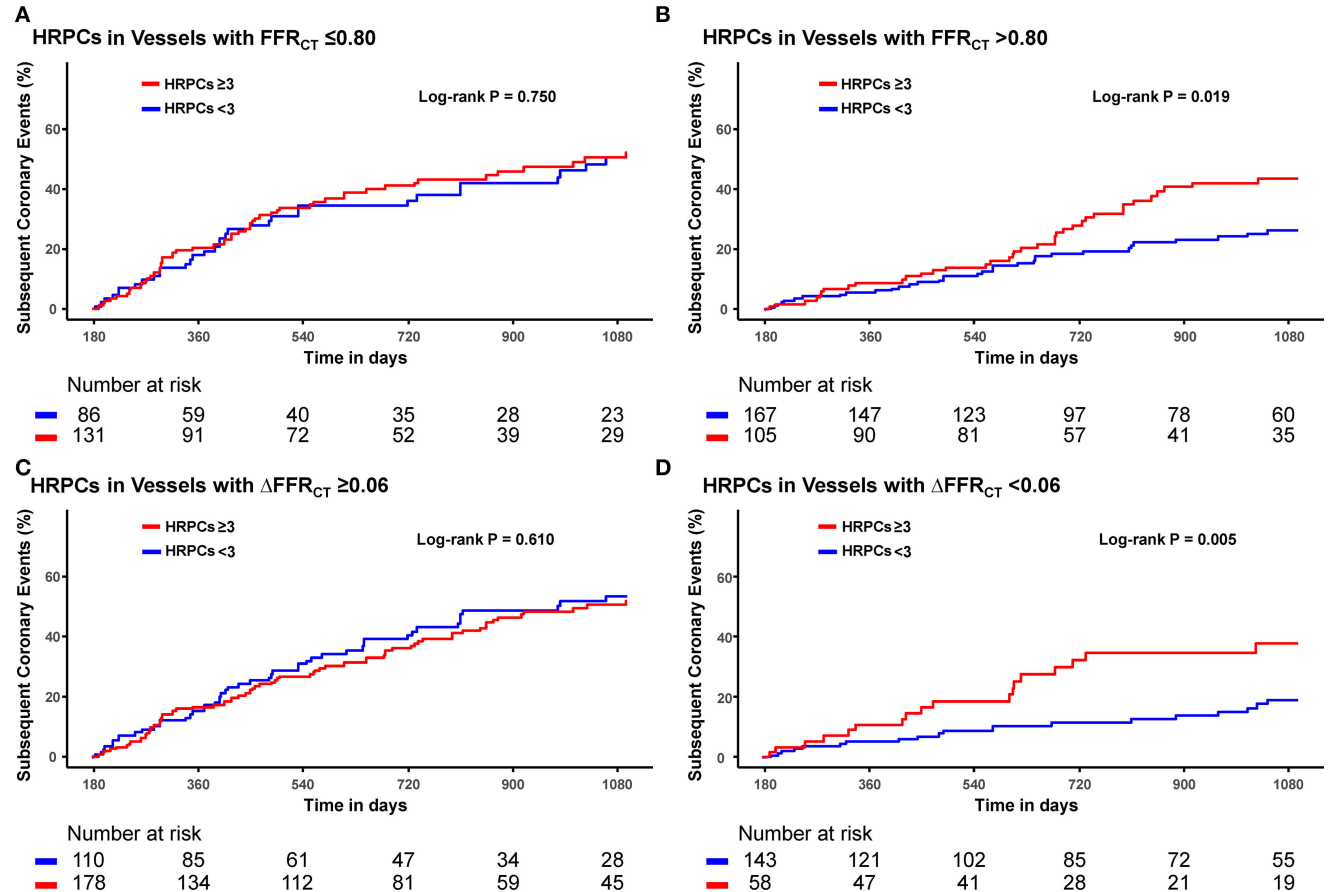
Differential prognostic impact of HRPCs for subsequent coronary events according to hemodynamic parameters. Differential prognostic impact of number of HRPCs of ≥3 is evaluated in vessels with **(A)** FFR_CT_ ≤0.80, **(B)** FFR_CT_ >0.80 **(C)** ΔFFR_CT_ ≥0.06, and **(D)** ΔFFR_CT_ <0.06. Abbreviations: FFR_CT_, fractional flow reserve by coronary computed tomography angiography; HRPCs, high-risk plaque characteristics.

**Figure 6:**
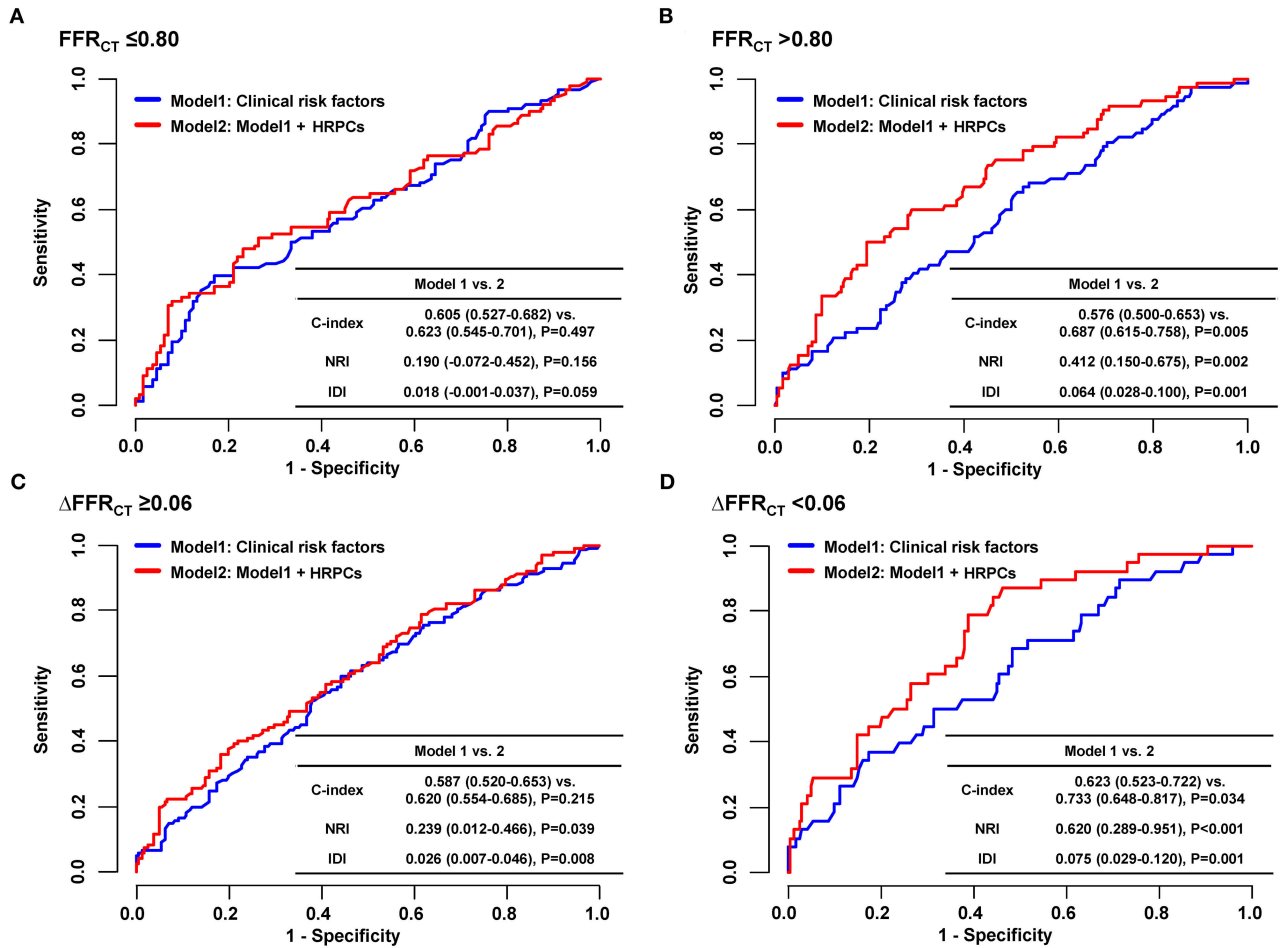
Incremental prognostic value of HRPCs for subsequent coronary events according to hemodynamic parameters. To assess incremental discriminatory and reclassification abilities of HRPCs in addition to clinical risk factors for subsequent coronary events, two models were constructed as follows: model 1: clinical risk factors (age, sex, hypertension, diabetes mellitus, dyslipidemia, chronic kidney disease, and current smoker) and model 2: clinical risk factors + HRPCs (low attenuation plaque, positive remodeling, napkin-ring sign, spotty calcification, minimal luminal area <4 mm^2^, or plaque burden ≥70%). Incremental discriminatory and reclassification abilities of HRPCs were evaluated in vessels with **(A)** FFR_CT_ ≤ 0.80, **(B)** FFR_CT_ > 0.80, **(C)** ΔFFR_CT_ ≥ 0.06, and **(D)** ΔFFR_CT_ < 0.06. FFR_CT_, fractional flow reserve by coronary computed tomography angiography; HRPCs, high-risk plaque characteristics; IDI, relative integrated discrimination improvement; NRI, category-free net reclassification index.

When the risk of subsequent coronary events was compared according to the number of HRPCs, a significantly higher risk of subsequent coronary events in vessels with HRPCs ≥ 3 than in vessels with HRPCs < 3 was observed only among vessels with negative FFR_CT_ or ΔFFR_CT_ (Figure 5). In addition, integration of HRPCs significantly improved discriminant and reclassification abilities for subsequent coronary events only in vessels with negative hemodynamic significance (FFR_CT_ > 0.80; 0.687 vs. 0.576, *P* = 0.005; NRI 0.412, *P* = 0.002; IDI 0.064, *P* = 0.001; ΔFFR_CT_ < 0.06; 0.733 vs. 0.623, *P* = 0.034; NRI 0.620, *P* < 0.001; IDI 0.075, *P* = 0.001) (Figure 6).

## Discussion

This study evaluated (1) the prognostic implications of CCTA-derived anatomic and hemodynamic plaque characteristics to predict subsequent coronary events, and (2) the differential prognostic implications of anatomic plaque characteristics according to the hemodynamic significance of CAD. The main findings are as follows: First, HRPCs and hemodynamic parameters showed higher discriminant abilities for subsequent coronary events than clinical risk factors, with ΔFFR_CT_ being the most powerful predictor. Second, HRPCs showed additive discriminant and reclassification abilities to clinical risk factors in the prediction of subsequent coronary events, which were further increased by adding hemodynamic parameters. Third, the prognostic impact of HRPCs was significant among vessels with negative hemodynamic significance (FFR_CT_ >0.80 or ΔFFR_CT_ <0.06) but not among those with positive hemodynamic significance (FFR_CT_ ≤ 0.80 or ΔFFR_CT_ ≥ 0.06).

### Risk Stratification for Subsequent Coronary Events Using CCTA

Identification of patients at increased risk of ACS who may benefit from intensified preventive measures has been a major challenge, and previous studies have consistently shown that prediction based on clinical risk factors is insufficient for adequate individual risk assessment (1). In contemporary practice, patients with suspected ischemic heart disease are commonly evaluated by noninvasive stress testing, which determines the need for invasive coronary angiography (8). However, the revascularization is not indicated in two-third of the cases sent for invasive coronary angiography due to anatomically or hemodynamically nonobstructive stenosis in epicardial coronary arteries (18). Previous studies demonstrated that those nonobstructive CAD could be accompanied by major adverse cardiovascular events including ACS (19), and the treatment strategy based on noninvasive stress testing did not significantly reduce the risk of ACS or ACS-related mortality compared with medical treatment alone (20, 21).

Conversely, recent trials showed that CCTA-based evaluation of CAD provided better risk stratification of high-risk patients and improved prognosis compared with standard care (22). Furthermore, multiple studies showed that CCTA-derived anatomic plaque characteristics (MLA, plaque burden, positive remodeling, low-attenuation plaque, napkin-ring sign, and spotty calcification) could provide additional information on the risk of ACS (4, 5). In addition, other studies presented the prognostic implications of CCTA-derived hemodynamic parameters. The prognostic value of FFR_CT_ has been confirmed for up to 5 years in several studies (10, 23, 24). Of note, in the ADVANCE registry, with the largest sample size (*N* = 5083), patients with negative FFR_CT_ > 0.80 showed a significantly lower risk of cardiac death or MI than those with FFR_CT_ ≤ 0.80 at 1 year (0.2% vs. 0.8%, *P* = 0.01) (10). The EMERALD study retrospectively evaluated 72 patients who had undergone CCTA before ACS events and compared CCTA-derived anatomic and hemodynamic plaque characteristics (FFR_CT_, ΔFFR_CT_, wall shear stress, and axial plaque stress) between the culprit and non-culprit vessels. In this study, plaques with adverse anatomic and hemodynamic characteristics had a significantly higher risk of ACS than those without (9). Despite the lack of a negative control group not presenting with ACS being a major limitation of the EMERALD study, the results supported the potential role of CCTA-based anatomic and hemodynamic plaque characteristics for better identification of high-risk patients. However, studies on the clinical role of the comprehensive evaluation of these features over clinical risk factors have been limited. Furthermore, there has been limited study, which evaluated the differential prognostic implications of CCTA-derived HRPCs according to the hemodynamic significance of CAD.

### Increased Predictability by CCTA-Derived Anatomic and Hemodynamic Plaque Evaluation

As with previous studies (1), discriminant ability of clinical risk factors to predict subsequent coronary events was limited, and the c-index of clinical risk factors was lower than that of most CCTA-derived anatomic plaque characteristics. Among the CCTA-derived anatomic plaque characteristics, plaque burden ≥70%, MLA <4 mm^2^, and low attenuation plaque showed higher discrimination abilities for subsequent coronary events than any individual clinical risk factor or other parameters of HRPCs. As with previous results from the 3V-FFR-FRIENDS study (5), the number of HRPCs showed a higher discrimination ability than either individual parameters of HRPCs or clinical risk factors.

More importantly, CFD-derived hemodynamic parameters were more predictive for subsequent coronary events than clinical risk factors or HRPCs. The three CFD-derived hemodynamic parameters evaluated in this study, namely, FFR_CT_, ΔFFR_CT_, and percent ischemic myocardial mass, represent different aspects of hemodynamic significance in the target vessel territory. The FFR_CT_ represents the severity of myocardial ischemia, whereas the percent ischemic myocardial mass represents the extent of myocardial ischemia. Furthermore, FFR_CT_ reflects cumulative hemodynamic deprivation of the entire target vessel, representing vessel-level significance, whereas ΔFFR_CT_ reflects the severity of local stenosis within the target vessel, representing lesion-level significance. Among these hemodynamic parameters, the discrimination abilities of FFR_CT_ and ΔFFR_CT_ were significantly higher than those of any clinical risk factors, individual HRPCs, or the number of HRPCs. These results support the contemporary practice guidelines that recommend treatment decisions based on the hemodynamic significance of the target lesion (8). Of note, ΔFFR_CT_ showed the highest discriminant ability for subsequent coronary events, suggesting that lesion-level hemodynamic significance may be the most important determinant of subsequent coronary events among other anatomic and hemodynamic parameters, including vessel-level FFR_CT_.

In the prediction of subsequent coronary events, CCTA-derived HRPCs and CFD-derived hemodynamic parameters showed incremental predictability when added to clinical risk factors. The final model with clinical risk factors, HRPCs, and hemodynamic parameters showed significantly increased discrimination and reclassification abilities. Considering that CCTA enables simultaneous assessment of both HRPCs and hemodynamic parameters without additional scans or invasive procedures, radiation exposure, or use of hyperemic agents, it would be a practical diagnostic and prognostic stratification tool for patients with suspected CAD who may need intensive medical treatment to prevent plaque progression and rupture. Further study is warranted to incorporate this concept into daily practice.

### Differential Prognostic Implications of HRPCs According to Hemodynamic Significance

Previous studies showed that there were associations among lesion severity, anatomic plaque characteristics, and hemodynamic lesion severity (2, 3, 5, 25). Similarly, we found that HRPCs were significantly associated with hemodynamic parameters, namely, FFR_CT_, ΔFFR_CT_, and percent ischemic myocardial mass. These associations can differ in each patient/lesion due to numerous patient- or lesion-specific parameters such as plaque content, presence of positive or negative remodeling, lesion location, or variation in coronary flow and microvascular function. Nevertheless, since current guidelines recommend treatment decisions based on hemodynamic significance but not the anatomic plaque characteristics, there has been an ongoing debate regarding the prognostic significance of HRPCs in lesions with negative hemodynamic significance. A recent 3V-FFR-FRIENDS study demonstrated that, among deferred vessels with FFR >0.80, those with ≥3 HRPCs showed a significantly higher risk of vessel-specific MI, revascularization, or cardiac death at 5 years compared with those with <3 HRPCs (5).

In this study, there was no additional role for HRPCs in predicting subsequent coronary events in vessels with positive hemodynamic significance (FFR_CT_ ≤ 0.80 or ΔFFR_CT_ < 0.06). Conversely, in vessels with negative hemodynamic significance, the presence of ≥3 HRPCs was independently associated with a higher risk of subsequent coronary events, and HRPCs showed an incremental discrimination ability when added to clinical risk factors. These results imply that additional evaluation of CCTA-based anatomic plaque characteristics in hemodynamically insignificant lesions might improve risk stratification in patients with CAD. Since ischemia-based imaging assessments with noninvasive stress tests cannot detect CAD without hemodynamic significance, CCTA-based anatomic plaque assessment would be helpful to select individuals at elevated risk for subsequent coronary events who could have been underdiagnosed by the stress tests. Furthermore, considering the differential prognostic implications of HRPCs according to hemodynamic significance, it should be further evaluated whether intensive medical therapy and/or preemptive PCI in hemodynamically insignificant lesions with HRPCs can induce stabilization of plaque characteristics (26) and eventually improve patient prognosis. Current ongoing trials (PREVENT [NCT02316886] and PROSPECT II [NCT02171065]) will help to clarify this issue.

### Limitations

Some limitations should be acknowledged. First, this study has limitations related to the observational design of the study. Consequently, confounding bias may occur due to measured and unmeasured variables. Second, only patients who underwent invasive coronary angiography 6–36 months after CCTA were included in the study, which may have caused selection bias. Therefore, the current results may not be generalized to an overall population who underwent CCTA. Further external validation is needed in future studies. Third, vessels that were not suitable for anatomic and hemodynamic plaque characteristics analyses were excluded. This may also have caused selection bias. In particular, vessels with severe calcification were excluded from the analysis as they were considered suboptimal for plaque characterization. Fourth, the decision to perform CCTA and PCI was left to the operator's discretion. Fifth, neither group received adequate preventive medication according to the current consensus. However, this might reflect real-world practice and show the natural course of the patients. Sixth, it should be noted that the overall accuracy of models to predict future ACS occurrences was not very high. This might reflect the complex nature of the underlying mechanism of ACS.

## Conclusion

**Figure 7:**
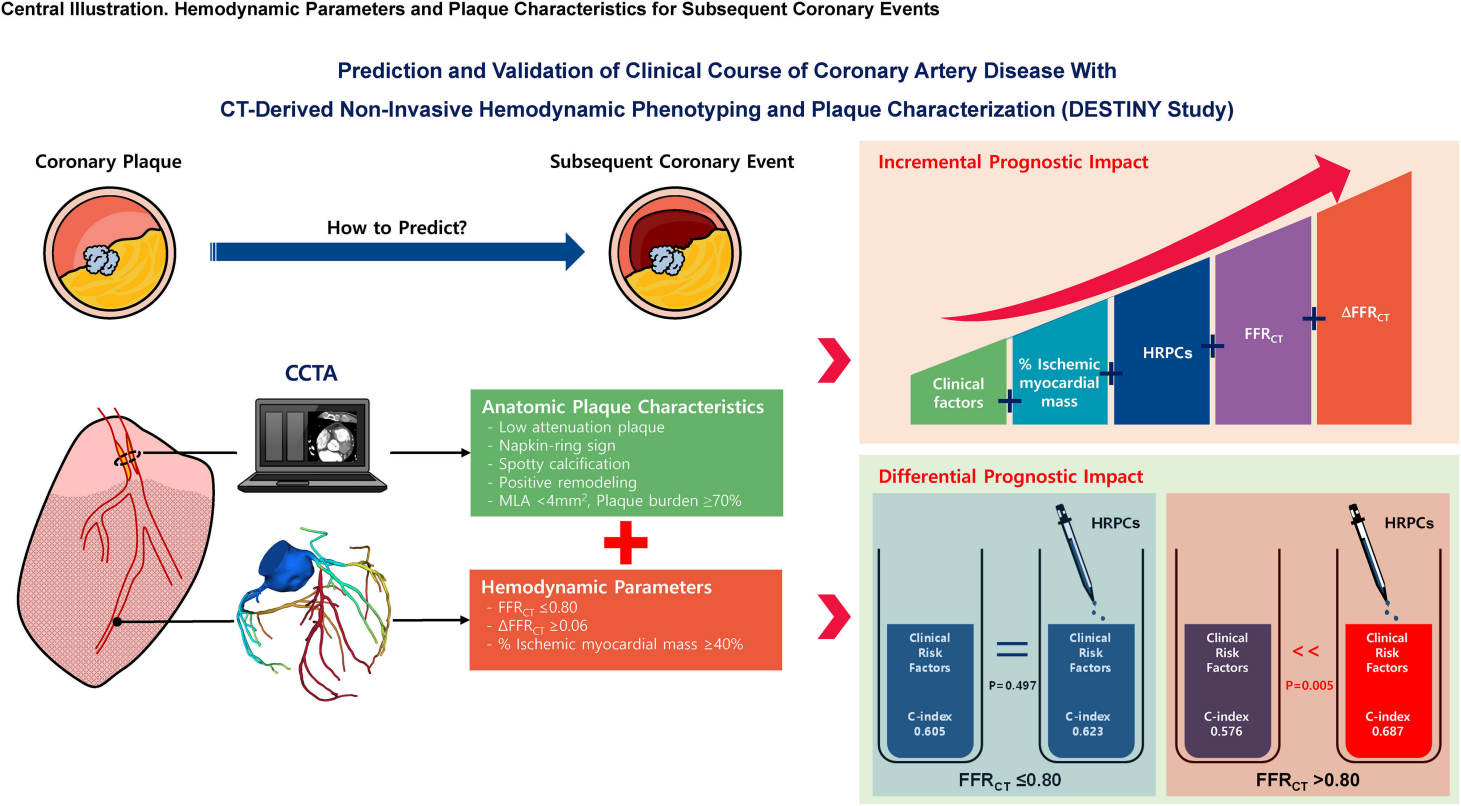
Hemodynamic parameters and plaque characteristics for subsequent coronary events. This study evaluated the prognostic implications of CCTA-derived HRPCs and hemodynamic parameters in the prediction of subsequent coronary events. Compared with clinical risk factors, HRPCs and hemodynamic parameters showed higher discriminant abilities for subsequent coronary events. HRPCs showed an additive discriminant ability to clinical risk factors, and hemodynamic parameters further increased discriminant ability for subsequent coronary events. Among vessels with negative FFR_CT_ (>0.80), adding HRPCs into clinical risk factors significantly increased discriminant and reclassification abilities for subsequent coronary events but not for vessels with positive FFR_CT_ (≤0.80). These results imply that additional evaluation of CCTA-based anatomic plaque characteristics in hemodynamically insignificant lesions might improve risk stratification in patients with CAD. FFR_CT_, fractional flow reserve by coronary computed tomography angiography; HRPCs, high-risk plaque characteristics; MLA, minimum lumen area.

In predicting subsequent coronary events, both HRPCs and hemodynamic parameters by CCTA allow for better prediction of subsequent coronary events than clinical risk factors alone. HRPCs provide incremental predictability than clinical risk factors among vessels with negative FFR_CT_ but not among vessels with positive FFR_CT_ (Figure 7).

## Data Availability Statement

The datasets presented in this article are not readily available because data cannot be shared publicly due to the privacy of individuals that participated in the study. The data will be shared on reasonable request to the corresponding author. Requests to access the datasets should be directed to JL, drone80@hanmail.net.

## Ethics Statement

The studies involving human participants were reviewed and approved by Samsung Medical Center. The patients/participants provided their written informed consent to participate in this study.

## Author Contributions

SL, DH, and JL: conception, design, analysis, interpretation of data, drafting and revising of the manuscript, and final approval of the manuscript submitted. ND, DS, KC, SK, HK, K-HJ, SH, KL, TP, JY, YS, S-HC, YC, H-CG, and JG: interpretation of data, revising of the manuscript, and final approval of the manuscript submitted. All authors contributed to the article and approved the submitted version.

## Conflict of Interest

JL received a Research Grant from St. Jude Medical (Abbott Vascular) and Philips Volcano. The remaining authors declare that the research was conducted in the absence of any commercial or financial relationships that could be construed as a potential conflict of interest.

## Publisher's Note

All claims expressed in this article are solely those of the authors and do not necessarily represent those of their affiliated organizations, or those of the publisher, the editors and the reviewers. Any product that may be evaluated in this article, or claim that may be made by its manufacturer, is not guaranteed or endorsed by the publisher.

## Abbreviations

ACS: acute coronary syndrome
CAD: coronary atherosclerotic disease
CCTA: coronary computed tomography angiography
CFD: computational fluid dynamics
FFR_CT_: fractional flow reserve by coronary computed tomography angiography
HR: hazard ratio
HRPCs: high-risk plaque characteristics
MI: myocardial infarction
PCI: percutaneous coronary intervention.
