# Supplementary material for: Anatomic and Hemodynamic Plaque Characteristics for Subsequent Coronary Events
Source: Front Cardiovasc Med. 2022 May 23;9:871450. doi: 10.3389/fcvm.2022.871450 (PMC9167998; doi:10.3389/fcvm.2022.871450)
Supplement: Supplementary file 1 [file Data_Sheet_1.docx]

**Supplementary Appendix**

**Hemodynamic Parameters and Plaque Characteristics for Subsequent Coronary Events**

Seung Hun Lee, MD, PhD^1†^, David Hong, MD^2†^, Neng Dai, MD^3^, Doosup Shin, MD^4^, and Joo Myung Lee, MD, MPH, PhD^2^

**Table of Contents**

- **Supplementary Methods**
- **Supplementary Tables**
- **Supplementary Figures and Figure Legends**
- **References**

**Supplementary Methods**

***Analysis of Hemodynamic Parameters in CCTA***

Hemodynamic parameters derived from CCTA were analyzed in a blinded fashion at a core laboratory (Zhongshan Hospital, Shanghai, China) using commercialized off-line software system (RuiXin-FFR, version 1.0, Raysight Medical, Shenzhen, China). First, three-dimensional anatomic computational models of the coronary tree were reconstructed from CCTA images. Second, patient-specific boundary conditions were obtained from the CCTA images. Third, hemodynamics parameters were acquired by CFD-based FFR_CT_ calculation.

***Three-dimensional model reconstruction***

Three-dimensional anatomic models of the coronary tree were reconstructed from CCTA images. First, coarse segmentation of the coronary tree was performed using Frangi filter and largest connected component detection.(1) Second, arterial centerlines were automatically extracted by region growing from the previous coarse segmentation.(2) Third, along the extracted centerlines, a new gradient-based method named “Coarse-to-Fine Subpixel” algorithm was used to generate lumen contours on the cross-sectional images. Finally, the surface model of the coronary tree was reconstructed by spline interpolation and lofting on all cross-sectional contours. Because pixel-level coarse coronary segmentation was insufficient for surface model reconstruction, a “Coarse-to-Fine Subpixel” algorithm for lumen contour was used to achieve more precise reconstructions (**Figure 1**). Specifically, we first extracted the cross-sectional image along the centerline and computed its gradient map. Next, we transferred the gradient image’s coordinate system to the polar coordinate system. K rays shot out from the center point at fixed angles, with each ray having fixed sample points with length L. We queued the lines and got a 2D polar image that size was K * L. In this image, we computed the K highest gradient magnitude points in all lines, and one smooth curve was fitted. Points in the smooth curve were related to the lumen contour points.

**Figure 1. “Coarse-to-Fine Subpixel” algorithm**


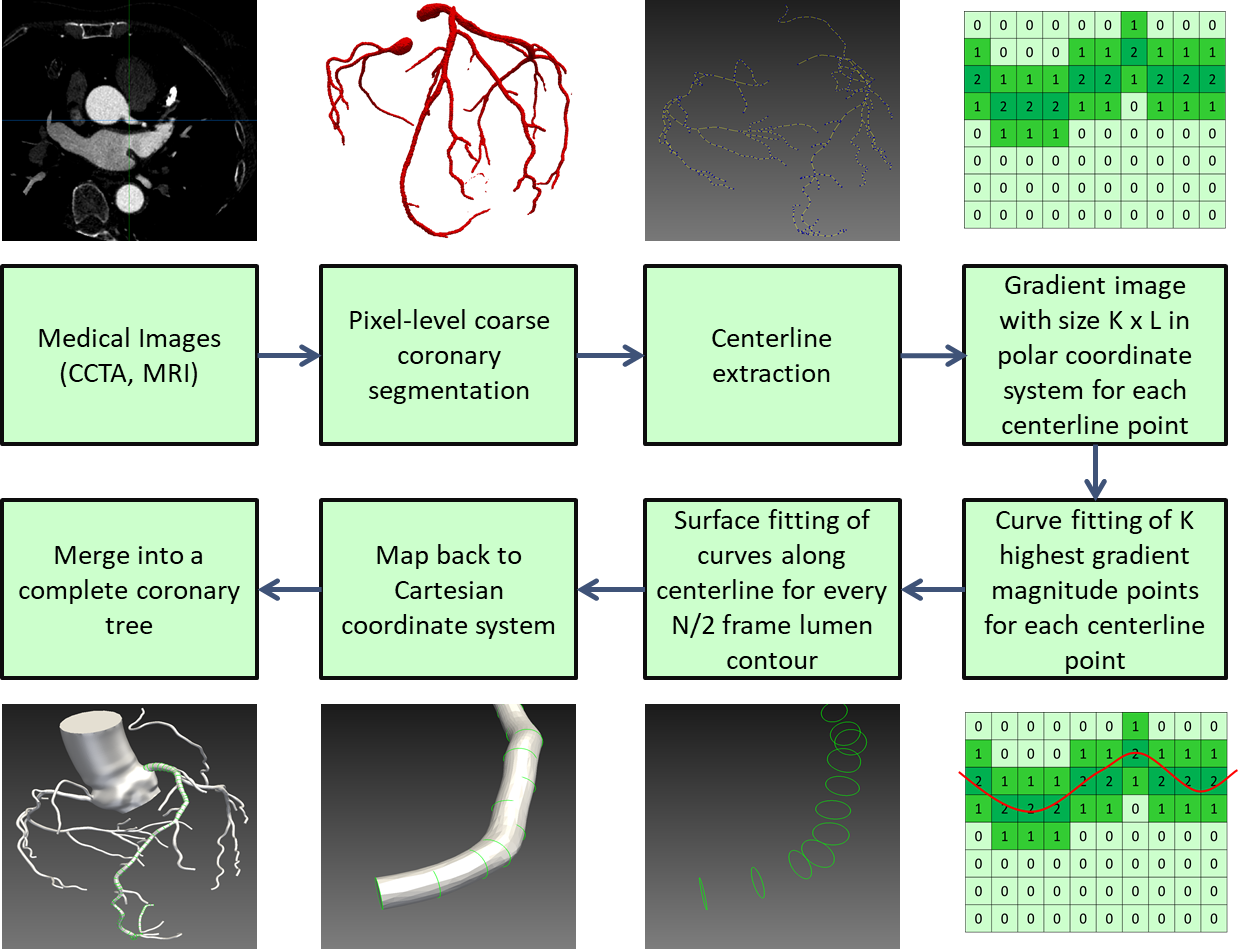


To achieve a stable and smooth lumen boundary, we mimicked the manner of radiologists, which refer to the nearest continuous multiple frames. Therefore, we extracted N nearest cross-sectional images to refine the N/2 frame lumen contour. After transferring each frame to polar coordinate system, the previous method was used to fit the curves of highest gradient. Next, we collected all points of the fitted curves to fit the surface by moving least squares (MLS) method. We then extracted the curve of the N/2 frame in the fitted surface to be the final curve, wherein each point in this curve was related to the lumen contour point in specific angle. Finally, we mapped these points back to the Cartesian coordinate system and fitted a closed boundary by Spline interpolation method. We used this closed contour to be the final lumen boundary.

For reconstructing the coronary tree model, precise lumen contours were generated at specified points along the coronary centerline. Typically, we extracted one lumen contour in each of the 7 centerline points. In addition, in some high curvature locations that the peaks of the curvature vs. centerline distance curve, we also generated lumen boundaries for avoiding abnormal shape.

***CFD-based Vessel-specific FFR_CT_ Calculation***

Patient-specific data, such as the volume of left ventricular myocardium, were also generated from the CCTA images. The total coronary flow rate at rest was derived from patient-specific data based on the modified algometric scaling laws (3,4) using coefficients that were optimized by using clinical data from an independent cohort. This cohort contains 103 patients with suspected coronary artery disease who were evaluated by CCTA. Given their coronary flow rate and myocardium volume, the coefficients were obtained by regression. Flow distribution among the 3 main coronary arteries was determined by the size of branches in the anatomic model using intraspecific scaling laws.(5) Total coronary flow rate at rest was scaled to account for hyperemia.(6) Blood was modeled as a Newtonian fluid. Incompressible Navier-Stokes equations were solved with a finite element method. Finally, FFR_CT_ values were obtained from the CFD solution.

**Supplementary Tables**

**Supplementary Table 1. Anatomic and Hemodynamic Plaque Characteristics of Vessels from Negative Control Cohort**

| Variables | **Vessels from Negative Control Cohort**  **(N=137)** |
| --- | --- |
| ***Interrogated Vessels*** |  |
| Left anterior descending artery | 55 (40.1) |
| Left circumflex artery | 41 (29.9) |
| Right coronary artery | 41 (29.9) |
| ***Anatomical severity*** |  |
| Diameter stenosis, % | 56.3 ± 18.6 |
| Area stenosis, % | 64.8 ± 19.7 |
| Minimum lumen area, mm^2^ | 1.7 ± 1.3 |
| Lesion length, mm | 21.5 ± 15.4 |
| ***Whole Vessel Tissue Characterization*** |  |
| Plaque burden, % | 81.4 ± 12.7 |
| Calcified volume, % | 4.7 ± 5.7 |
| Maximum calcified area, % | 27.9 ± 27.9 |
| Intra-plaque hemorrhage volume, mm^3^ | 4.6 ± 7.4 |
| Maximum intra-plaque hemorrhage area, mm^2^ | 0.81 ± 1.12 |
| Lipid-rich necrotic core volume, mm^3^ | 1.7 ± 4.0 |
| Maximum lipid-rich necrotic core area, mm^2^ | 0.44 ± 0.80 |
| Perivascular adipose tissue volume, % | 28.6 ± 13.0 |
| Vessel length, mm | 78.8 ± 37.6 |
| ***Target Plaque Tissue Characterization*** |  |
| Plaque burden, % | 81.2 ± 12.5 |
| Calcified volume, % | 12.6 ± 7.8 |
| Maximum calcified area, % | 36.3 ± 27.1 |
| Intra-plaque hemorrhage volume, mm^3^ | 1.8 ± 2.6 |
| Maximum intra-plaque hemorrhage area, mm^2^ | 0.48 ± 0.58 |
| Lipid-rich necrotic core volume, mm^3^ | 1.2 ± 2.6 |
| Maximum lipid-rich necrotic core area, mm^2^ | 0.37 ± 0.68 |
| ***High-Risk Plaque Characteristics*** |  |
| Low attenuation plaque | 28 (20.4) |
| Positive remodeling | 55 (40.1) |
| Napkin-ring sign | 28 (20.4) |
| Spotty calcification | 98 (71.5) |
| Minimum lumen area <4mm^2^ | 116 (84.7) |
| Plaque burden at lumen area ≥70% | 4 (2.9) |
| Number of HRPC | 2.4 ± 1.4 |
| Number of HRPC ≥3 | 73 (53.3) |
| ***Hemodynamic Plaque Characteristics*** |  |
| FFR_CT_ | 0.81 ± 0.11 |
| ∆FFR_CT_ | 0.09 ± 0.09 |
| Ischemia myocardial mass, % | 28.6 ± 19.1 |

**Supplementary Table 2. Diagnostic Performance of Hemodynamic Index and Plaque Characteristics**

|  | **FFR_CT_** | **∆FFR_CT_** | **% Ischemic Myocardial Mass** | **Number of HRPC** |
| --- | --- | --- | --- | --- |
| **Cut-off value** | **0.80** | **0.06** | **40** | **3** |
| Sensitivity, % | 55.0 | 76.3 | 32.5 | 57.5 |
| Specificity, % | 60.8 | 49.5 | 75.1 | 56.2 |
| Positive predictive value, % | 40.6 | 42.4 | 38.8 | 39.0 |
| Negative predictive value, % | 73.5 | 81.1 | 69.6 | 73.1 |
| Diagnostic accuracy, % | 58.9 | 58.3 | 61.1 | 56.6 |

Abbreviations: FFR_CT_, fractional flow reserve by coronary computed tomography angiography; HRPC, high-risk plaque characteristics.

**Supplementary Table 3. Anatomic Plaque Characteristics According to Fractional Flow Reserve (Per-vessel)**

| **Variables** | **Positive FFR_CT_ (≤0.80)**  **(N=217)** | | | **Negative FFR_CT_ (>0.80)**  **(N=272)** | | |
| --- | --- | --- | --- | --- | --- | --- |
|  | **Vessels with Subsequent Coronary Event (N=88)** | **Vessels without Subsequent Coronary Event (N=129)** | **P value** | **Vessels with Subsequent Coronary Event (N=72)** | **Vessels without Subsequent Coronary Event (N=200)** | **P value** |
| ***Anatomical severity*** | | | | | | |
| Diameter stenosis, % | 61.5 ± 17.8 | 60.2 ± 18.2 | 0.465 | 54.4 ± 18.2 | 47.6 ± 16.0 | 0.003 |
| Area stenosis, % | 69.4 ± 17.2 | 68.3 ± 18.9 | 0.417 | 62.9 ± 20.6 | 56.9 ± 18.3 | 0.024 |
| Minimum lumen area, mm^2^ | 1.2 ± 1.0 | 1.3 ± 1.0 | 0.699 | 1.9 ± 1.5 | 2.3 ± 1.5 | 0.035 |
| Lesion length, mm | 25.0 ± 17.7 | 25.5 ± 19.0 | 0.879 | 23.4 ± 15.8 | 18.7 ± 15.3 | 0.085 |
| ***Whole Vessel Tissue Characterization*** | | | | | | |
| Plaque burden area, % | 86.0 ± 9.9 | 84.4 ± 10.6 | 0.229 | 79.1 ± 12.2 | 75.4 ± 13.1 | 0.041 |
| Calcified volume, % | 4.9 ± 5.7 | 6.0 ± 6.7 | 0.210 | 3.7 ± 4.8 | 3.2 ± 4.4 | 0.491 |
| Maximum calcified area, % | 26.0 ± 20.2 | 30.2 ± 27.7 | 0.193 | 22.3 ± 21.9 | 21.6 ± 18.8 | 0.804 |
| Intra-plaque hemorrhage volume, mm^3^ | 5.0 ± 7.5 | 4.2 ± 6.9 | 0.379 | 4.4 ± 12.4 | 3.8 ± 6.1 | 0.713 |
| Maximum intra-plaque hemorrhage area, mm^2^ | 0.86 ± 1.00 | 0.77 ± 1.11 | 0.515 | 0.64 ± 0.78 | 0.67 ± 0.90 | 0.791 |
| Lipid-rich necrotic core volume, mm^3^ | 2.6 ± 6.3 | 1.7 ± 4.1 | 0.184 | 0.9 ± 2.3 | 0.9 ± 2.3 | 0.906 |
| Maximum lipid-rich necrotic core area, mm^2^ | 0.51 ± 0.83 | 0.39 ± 0.78 | 0.262 | 0.29 ± 0.68 | 0.27 ± 0.51 | 0.827 |
| Perivascular adipose tissue volume, % | 32.6 ± 12.5 | 29.1 ± 12.8 | 0.053 | 30.8 ± 12.2 | 29.5 ± 12.5 | 0.474 |
| Vessel length, mm | 78.5 ± 33.4 | 71.7 ± 35.5 | 0.142 | 70.9 ± 34.0 | 77.5 ± 36.6 | 0.159 |
| ***Target Plaque Tissue Characterization*** | | | | | | |
| Plaque burden area, % | 87.6 ± 9.7 | 85.6 ± 11.0 | 0.258 | 82.2 ± 9.5 | 76.8 ± 12.2 | 0.003 |
| Calcified volume, % | 11.2 ± 8.3 | 13.3 ± 9.1 | 0.147 | 9.6 ± 8.0 | 11.1 ± 7.1 | 0.258 |
| Maximum calcified area, % | 31.7 ± 19.0 | 37.5 ± 27.6 | 0.113 | 30.3 ± 22.0 | 30.7 ± 14.5 | 0.908 |
| Intra-plaque hemorrhage volume, mm^3^ | 3.2 ± 5.6 | 2.4 ± 3.9 | 0.314 | 2.9 ± 7.0 | 1.6 ± 3.2 | 0.275 |
| Maximum intra-plaque hemorrhage area, mm^2^ | 0.83 ± 1.09 | 0.59 ± 0.78 | 0.145 | 0.65 ± 0.93 | 0.47 ± 0.76 | 0.250 |
| Lipid-rich necrotic core volume, mm^3^ | 2.5 ± 6.4 | 1.3 ± 3.2 | 0.171 | 0.4 ± 1.1 | 0.7 ± 1.7 | 0.186 |
| Maximum lipid-rich necrotic core area, mm^2^ | 0.47 ± 0.83 | 0.35 ± 0.67 | 0.333 | 0.19 ± 0.42 | 0.24 ± 0.50 | 0.531 |
| ***High-Risk Plaque Characteristics*** | | | | | | |
| Low attenuation plaque | 26 (29.5) | 30 (23.3) | 0.378 | 20 (27.8) | 26 (13.0) | 0.007 |
| Positive remodeling | 42 (47.7) | 65 (50.4) | 0.805 | 33 (45.8) | 71 (35.5) | 0.16 |
| Napkin-ring sign | 17 (19.3) | 27 (20.9) | 0.906 | 10 (13.9) | 26 (13.0) | >0.999 |
| Spotty calcification | 67 (76.1) | 95 (73.6) | 0.798 | 46 (63.9) | 126 (63.0) | >0.999 |
| Minimum lumen area <4mm^2^ | 80 (90.9) | 117 (90.7) | >0.999 | 60 (83.3) | 144 (72.0) | 0.081 |
| Plaque burden at lumen area ≥70% | 9 (10.2) | 9 (7.0) | 0.547 | 7 (9.7) | 4 (2.0) | 0.012 |
| Number of HRPC | 2.7 ± 1.4 | 2.7 ± 1.4 | 0.390 | 2.4 ± 1.5 | 2.0 ± 1.4 | 0.018 |
| Number of HRPC ≥3 | 55 (62.5) | 76 (58.9) | 0.697 | 37 (51.4) | 68 (34.0) | 0.014 |

Data are presented as mean ± standard deviation, or number (%).

Abbreviations: FFR_CT_, fractional flow reserve by coronary computed tomography angiography; HRPC, high-risk plaque characteristics.

**Supplementary Table 4.** **Anatomic Plaque Characteristics According to ∆FFR_CT_ (Per-vessel)**

| **Variables** | **Positive ∆FFR_CT_ (≥0.06)**  **(N=288)** | | | **Negative ∆FFR_CT_ (<0.06)**  **(N=201)** | | |
| --- | --- | --- | --- | --- | --- | --- |
|  | **Vessels with Subsequent Coronary Event (N=122)** | **Vessels without Subsequent Coronary Event (N=166)** | **P value** | **Vessels with Subsequent Coronary Event (N=38)** | **Vessels without Subsequent Coronary Event (N=163)** | **P value** |
| ***Anatomical severity*** | | | | | | |
| Diameter stenosis, % | 60.7 ± 19.0 | 58.7 ± 18.2 | 0.254 | 50.9 ± 13.2 | 46.1 ± 15.3 | 0.063 |
| Area stenosis, % | 68.6 ± 19.4 | 67.6 ± 18.8 | 0.556 | 60.2 ± 16.2 | 54.9 ± 17.8 | 0.113 |
| Minimum lumen area, mm^2^ | 1.4 ± 1.3 | 1.4 ± 1.0 | 0.955 | 1.9 ± 1.1 | 2.4 ± 1.5 | 0.008 |
| Lesion length, mm | 25.8 ± 17.7 | 25.7 ± 18.1 | 0.963 | 17.7 ± 10.8 | 16.7 ± 14.9 | 0.740 |
| ***Whole Vessel Tissue Characterization*** | | | | | | |
| Plaque burden area, % | 85.0 ± 11.3 | 84.6 ± 10.9 | 0.802 | 76.5 ± 9.8 | 73.0 ± 12.3 | 0.055 |
| Calcified volume, % | 4.9 ± 5.6 | 5.7 ± 6.4 | 0.273 | 2.6 ± 3.6 | 2.8 ± 4.2 | 0.703 |
| Maximum calcified area, % | 26.2 ± 20.3 | 29.6 ± 26.3 | 0.211 | 18.5 ± 22.1 | 20.2 ± 18.0 | 0.651 |
| Intra-plaque hemorrhage volume, mm^3^ | 5.7 ± 11.2 | 4.6 ± 6.6 | 0.371 | 1.9 ± 2.7 | 3.3 ± 6.2 | 0.025 |
| Maximum intra-plaque hemorrhage area, mm^2^ | 0.86 ± 0.99 | 0.87 ± 1.15 | 0.921 | 0.46 ± 0.54 | 0.54 ± 0.74 | 0.482 |
| Lipid-rich necrotic core volume, mm^3^ | 2.2 ± 5.6 | 1.7 ± 3.8 | 0.373 | 0.8 ± 2.5 | 0.7 ± 2.2 | 0.924 |
| Maximum lipid-rich necrotic core area, mm^2^ | 0.45 ± 0.76 | 0.41 ± 0.73 | 0.641 | 0.28 ± 0.80 | 0.22 ± 0.50 | 0.642 |
| Perivascular adipose tissue volume, % | 32.7 ± 11.8 | 29.6 ± 12.4 | 0.031 | 28.8 ± 13.8 | 29.1 ± 13.0 | 0.890 |
| Vessel length, mm | 77.2 ± 33.9 | 79.3 ± 34.9 | 0.610 | 68.6 ± 33.0 | 70.9 ± 37.2 | 0.709 |
| ***Target Plaque Tissue Characterization*** | | | | | | |
| Plaque burden area, % | 86.8 ± 9.7 | 85.3 ± 11.0 | 0.334 | 79.4 ± 8.9 | 74.8 ± 11.7 | 0.048 |
| Calcified volume, % | 10.8 ± 8.3 | 13.1 ± 9.0 | 0.069 | 9.5 ± 7.9 | 10.8 ± 6.7 | 0.497 |
| Maximum calcified area, % | 31.5 ± 19.5 | 36.3 ± 26.1 | 0.120 | 29.3 ± 23.3 | 30.4 ± 13.0 | 0.831 |
| Intra-plaque hemorrhage volume, mm^3^ | 3.5 ± 6.6 | 2.6 ± 4.1 | 0.293 | 1.2 ± 2.7 | 1.2 ± 2.6 | 0.991 |
| Maximum intra-plaque hemorrhage area, mm^2^ | 0.84 ± 1.10 | 0.65 ± 0.87 | 0.212 | 0.38 ± 0.54 | 0.37 ± 0.60 | 0.891 |
| Lipid-rich necrotic core volume, mm^3^ | 2.0 ± 5.6 | 1.3 ± 3.1 | 0.321 | 0.3 ± 0.7 | 0.6 ± 1.5 | 0.274 |
| Maximum lipid-rich necrotic core area, mm^2^ | 0.40 ± 0.75 | 0.36 ± 0.65 | 0.699 | 0.19 ± 0.46 | 0.20 ± 0.48 | 0.934 |
| ***High-Risk Plaque Characteristics*** | | | | | | |
| Low attenuation plaque | 39 (32.0) | 44 (26.5) | 0.379 | 7 (18.4) | 12 (7.4) | 0.073 |
| Positive remodeling | 59 (48.4) | 92 (55.4) | 0.286 | 16 (42.1) | 44 (27.0) | 0.102 |
| Napkin-ring sign | 24 (19.7) | 37 (22.3) | 0.696 | 3 (7.9) | 16 (9.8) | 0.955 |
| Spotty calcification | 91 (74.6) | 123 (74.1) | >0.999 | 22 (57.9) | 98 (60.1) | 0.945 |
| Minimum lumen area <4mm^2^ | 107 (87.7) | 147 (88.6) | 0.971 | 33 (86.8) | 114 (69.9) | 0.056 |
| Plaque burden at lumen area ≥70% | 14 (11.5) | 10 (6.0) | 0.150 | 2 (5.3) | 3 (1.8) | 0.521 |
| Number of HRPC | 2.7 ± 1.4 | 2.7 ± 1.4 | 0.748 | 2.2 ± 1.4 | 1.8 ± 1.3 | 0.035 |
| Number of HRPC ≥3 | 74 (60.7) | 104 (62.7) | 0.825 | 18 (47.4) | 40 (24.5) | 0.009 |

Data are presented as mean ± standard deviation, or number (%).

Abbreviations: FFR_CT_, fractional flow reserve by coronary computed tomography angiography; HRPC, high-risk plaque characteristics.

**Supplementary Figures**

**Supplementary Figure 1. Association between HRPC with FFR_CT_, ∆FFR_CT_, % Ischemic Myocardial Mass and Clinical Risk Factors**

Number of HRPC was associated with (A) number of clinical risk factors, (B) FFR_CT_, (C) **∆**FFR_CT_, and (D) % Ischemic Myocardial Mass.

Abbreviations: FFR_CT_, fractional flow reserve by coronary computed tomography angiography; HRPC, high-risk plaque characteristics.

**Supplementary Figure 2. Comparison of Prediction Models for Subsequent Coronary Events**

Incremental prognostic value of anatomic and hemodynamic plaque characteristics in addition to clinical risk factors were evaluated.

Models are constructed as follows.

Model 1: clinical risk factors

Model 2: model 1 + individual component of HRPC

Model 3: model 2 + hemodynamic parameters

Components of clinical risk factors, hemodynamic parameters, and HRPC are as follows: Clinical risk factors: age, sex, hypertension, diabetes, dyslipidemia, chronic kidney disease, current smoker; HRPC: low attenuation plaque, positive remodeling, napkin-ring sign, spotty calcification, minimum lumen area < 4mm^2^, plaque burden at lumen area ≥70%; Hemodynamic parameters: % ischemic myocardial mass, FFR_CT_, ∆FFR_CT_.

Abbreviations: FFR_CT_, fractional flow reserve by coronary computed tomography angiography; HRPC, high-risk plaque characteristics.

**
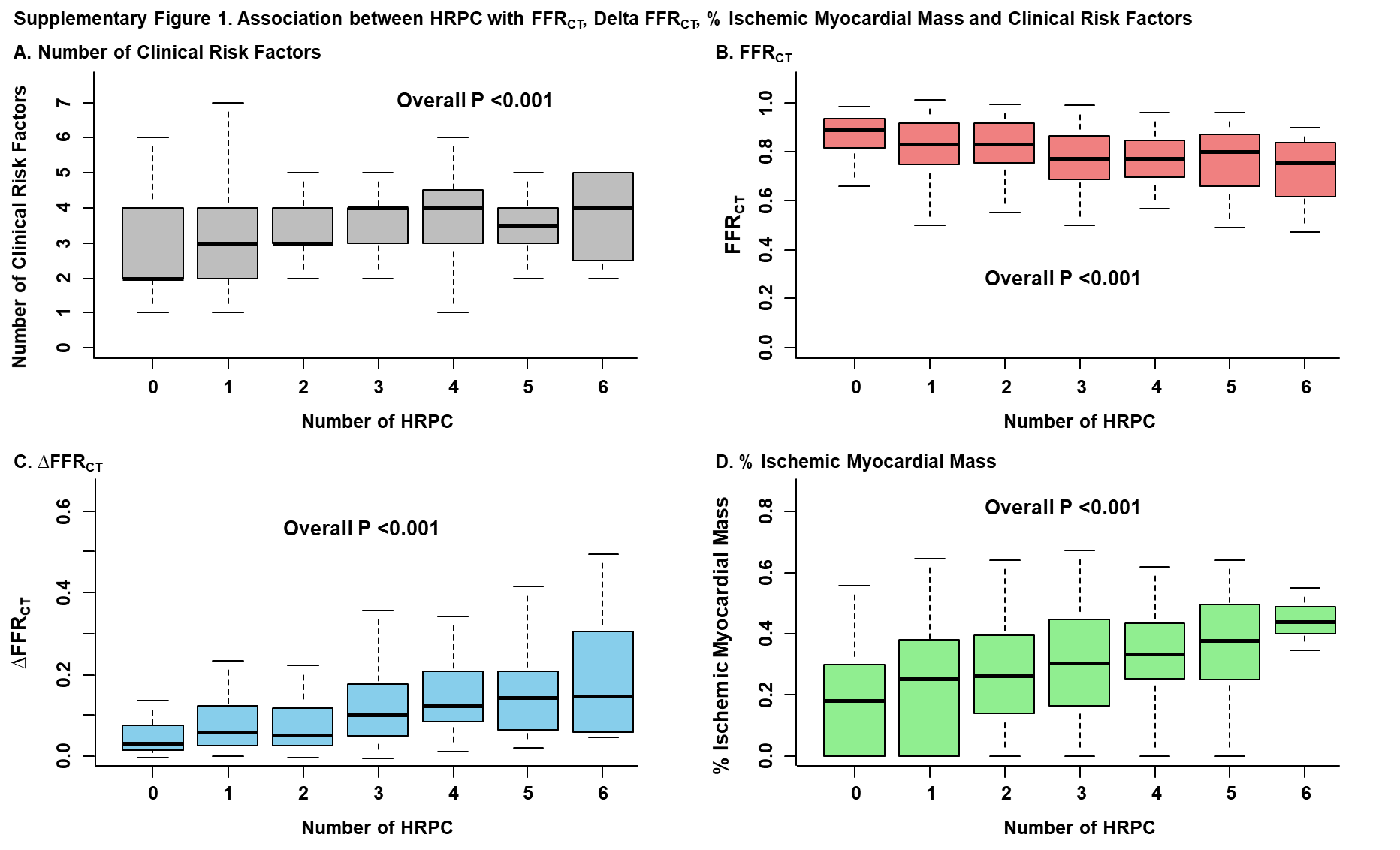
**

**
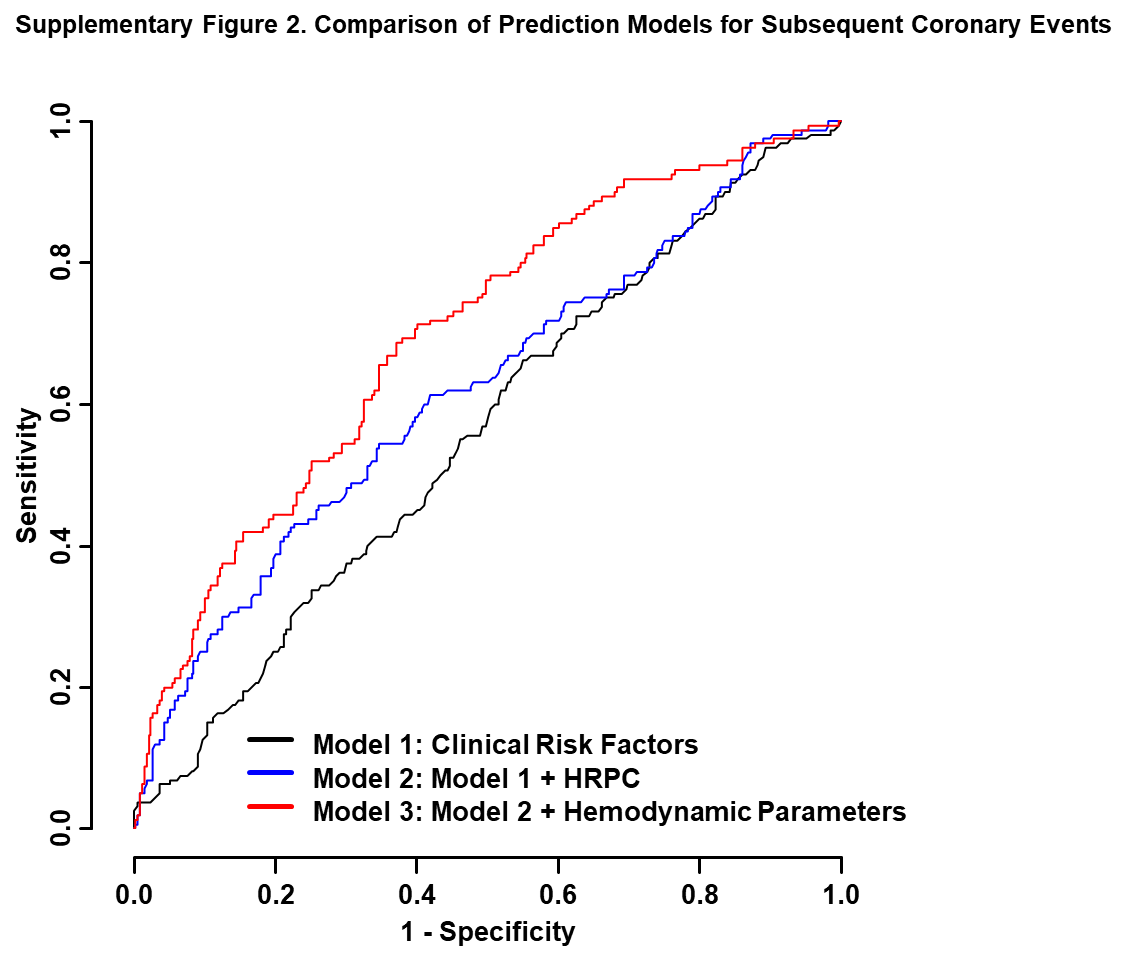
**

**References**

**1.** Frangi AF, Niessen WJ, Vincken KL, Viergever MA. Multiscale vessel enhancement filtering. Berlin, Heidelberg: Springer Berlin Heidelberg, 1998:130-7.

**2.** Reynisson PJ, Scali M, Smistad E, et al. Airway Segmentation and Centerline Extraction from Thoracic CT - Comparison of a New Method to State of the Art Commercialized Methods. PLoS One 2015;10:e0144282.

**3.** Murray CD. The Physiological Principle of Minimum Work: I. The Vascular System and the Cost of Blood Volume. Proc Natl Acad Sci U S A 1926;12:207-14.

**4.** Choy JS, Kassab GS. Scaling of myocardial mass to flow and morphometry of coronary arteries. J Appl Physiol (1985) 2008;104:1281-6.

**5.** Huo Y, Kassab GS. Intraspecific scaling laws of vascular trees. J R Soc Interface 2012;9:190-200.

**6.** Smits P, Thien T. Effects of adenosine on human coronary arterial circulation. Circulation 1991;84:2208-10.

**7.** Kashiwagi M, Kitabata H, Tanaka A, et al. Combination of Lesion Stenosis and Myocardial Supply Area Assessed by Coronary Computed Tomography Angiography for Prediction of Myocardial Ischemia. Int Heart J 2019;60:1238-44.

**8.** Ihdayhid AR, Norgaard BL, Achenbach S, et al. Ischemic Myocardial Burden Subtended by Computed Tomography-Derived Fractional Flow Reserve (APPROACH(FFRCT)): An Exploratory Analysis on Diagnostic Performance. JACC Cardiovasc Imaging 2020;13:2264-7.

**9.** West GB, Brown JH, Enquist BJ. A general model for the origin of allometric scaling laws in biology. Science 1997;276:122-6.

**10.** Kim HY, Doh JH, Lim HS, et al. Identification of Coronary Artery Side Branch Supplying Myocardial Mass That May Benefit From Revascularization. JACC Cardiovasc Interv 2017;10:571-81.
